# *S*-values for bone marrow dosimetry in preclinical radiopharmaceutical studies with rodents

**DOI:** 10.1186/s40658-025-00752-5

**Published:** 2025-07-08

**Authors:** Mohammed Obaid, Arman Rahmim, William P. Segars, Julia Brosch-Lenz, Carlos Uribe

**Affiliations:** 1 Department of Integrative Oncology, BC Cancer Research Institute, Vancouver, Canada; 2 Department of Radiology, University of British Columbia, Vancouver, Canada; 3 Department of Physics and Astronomy, University of British Columbia, Vancouver, Canada; 4 Carl E. Ravin Advanced Imaging Laboratories, Department of Radiology, Duke University School of Medicine, Durham, NC USA; 5 Department of Nuclear Medicine, School of Medicine, and Klinikum Rechts Der Isar, Technical University of Munich, Munich, Germany

**Keywords:** *S*-values, Bone marrow dosimetry, GATE, Monte Carlo, MOBY phantom

## Abstract

**Background:**

Development of novel radiopharmaceuticals involves dosimetry calculations to validate safety and aid with selection of those that should be translated into the clinical environment. Dosimetry is critical for limiting radiation damage to organs at risk. The bone marrow is a limiting organ in radiopharmaceutical therapies (RPTs) for metastatic prostate cancer, for example, but there is room for improvement of bone marrow dosimetry in preclinical studies. Bone marrow *S*-values for Lutetium-177 (^177^Lu) in rodents have been published but they have not included tumor xenografts inoculated in the shoulder, which is how radiopharmaceuticals are often tested. Here, we aim at performing Monte Carlo simulations on digital mice phantoms including tumor xenografts, and to determine new bone marrow S-values that can potentially improve our understanding of the effect of RPTs in blood cells.

**Methods:**

*S*-values for ^177^Lu were simulated in the 4D Mouse Whole Body (MOBY) phantom, a hybrid voxel-based mouse model, using GATE v9.3 MC toolkit. Two phantoms of different resolutions and equal mass were created. 3D dose distributions were simulated and the corresponding organ to organ *S*-values were calculated. The resulting *S*-values were validated against reference values from OLINDA v2.2.3. Later, tumours of varying sizes were placed in the left shoulder and tumour-to-organ *S*-values were calculated from MC simulations with a ^177^Lu source placed uniformly in these tumours.

**Results:**

The phantoms simulated here differed from the OLINDA phantom in both organ mass and geometry for many tissues; *S*-value deviations from OLINDA were correlated with these differences, as reported in previous studies, and ranged from 2% for the kidney self-dose in the higher resolution (HR) phantom to 477% for *S*(skeleton←spleen) in the lower resolution (LR) phantom. *S*-values were simulated for the bone marrow in both phantoms; cross-dose values were greatest from the skeleton, brain, and lungs, while cross-doses from the simulated tumours were approximately constant at 3 × 10^–15^ Gy Bq^−1^ s^−1^ across all tumour sizes. The components of the skeleton receiving the greatest tumour cross-doses from the tumours were the spine, skull and marrow. *S*-values targeting the bone marrow were compared to similar values from a previous study, whose phantom differed in tissue composition—discrepancies ranged from 6% for S(BM←kidneys) at LR to 87% for *S*(BM←BM) at HR. In general, relative uncertainty in dose and dose factor deposited from one tissue to another was inversely proportional to the corresponding *S*-value magnitude, and lower uncertainties were yielded from simulations in the LR, large-voxel phantom.

**Conclusion:**

Using the MOBY digital mouse phantom, we simulated bone marrow *S*-values for ^177^Lu. We hope these values help researchers perform preclinical dosimetry in rodents including bone marrow and tumor xenografts and facilitate the translation of novel radiopharmaceuticals.

**Supplementary Information:**

The online version contains supplementary material available at 10.1186/s40658-025-00752-5.

## Introduction

Radiopharmaceutical therapy (RPT) has shown promise in the treatment of prostate cancer [1–4] and neuroendocrine tumours [1, 5, 6]*.* In particular, the recent FDA approval of ^177^Lu-labeled radiopharmaceuticals has raised interest in theranostics and RPTs [3, 4, 7–10]*.* Novel radiopharmaceuticals are continually developed, but regulatory requirements mandate that they undergo rigorous testing in animal models during the preclinical phase before advancing to clinical trials [11, 12]. The aim of a radiopharmaceutical for RPTs is to deliver cytotoxic radiation to the tumour, while minimizing toxicity to healthy tissues. Dosimetry is used to quantify the energy deposited per unit mass of tissue, and to correlate with biological effects of radiation [9, 13, 14].

Dosimetry requires the determination of the time-integrated activity, which depends on biological factors, and the physical properties of the radioisotope that together determine the *S*-values (defined as the absorbed dose in a target tissue per unit cumulated activity in a source tissue). *S*-values are estimated using numerical methods like Monte Carlo (MC) simulations in anthropomorphic or animal digital phantoms. *S*-values can be tabulated for a source-target combination for a specific anatomical configuration and radionuclide [15, 16].

The 4D Mouse Whole Body (MOBY) phantom developed by Segars et al. is a realistic hybrid mouse model, commonly used for dosimetry in preclinical studies (Table 1)[17]. Based on a 33 g C57BL/6 mouse, the phantom uses non-uniform rational B-spline (NURBS) surfaces to define organs and body contours in a flexible manner that allows for modelling of a customized mouse anatomy, including organ motion or deformation and lesion simulation. MOBY was used by Keenan et al. for MC simulations of specific absorbed fractions (SAFs) and dose factors for the Radiation Dose Assessment Resource (RADAR) database [18]. Stabin and Siegel included *S*-values calculated using the MOBY phantom in the OLINDA/EXM v2.0 code [19, 20]. MOBY has been used by groups to characterize the dependence of *S*-values on anatomical variables. Xie et al. performed MC dose simulations to investigate the influence of organ mass and size [21]. Kostou et al. simulated organ-organ *S*-values in the MOBY phantom characterizing their dependence on organ mass scaling as well as whole-body *S*-values for a range of known activity biodistributions of preclinical radiotracers [22]. Elsewhere, groups have used MOBY to compare* S*-values between phantoms; Larsson et al. compared MOBY to earlier mathematical models based on ellipsoids and cylinders, while Mauxion et al. compared their own MOBY-simulated values to those from RADAR, finding variations between two different phantoms produced by the same software [23, 24].

A growing area of interest is simulation of S-values for the bone marrow (BM). BM is an organ at risk in some RPTs such as those targeting prostate cancer, due to its intrinsic radiosensitivity and the potential for cytotoxicity to cause marrow suppression, increasing the risk of conditions such as lymphocytopenia, leukopenia, and anaemia [2, 25–28]. Additionally, during clinical trials for ^177^Lu peptide receptor radionuclide therapy targeting neuroendocrine tumours, increased BM absorbed dose per treatment cycle has been correlated with decreases in platelet counts [5]. The structure and function of BM, particularly in the context of radiation dosimetry, has been characterized in greater detail by Bolch et al. [29] and Pichardo et al. [30]. The risk of toxicity may increase for those newer radiopharmaceuticals being developed with albumin binders, causing increased circulation in the blood. Thus, BM dosimetry has become a subfield with models developed to accurately estimate radiation dose to this region, both in humans and mice (Table 1) [26, 31, 32]. The first such models for humans were developed by Spiers for beta emitters, leading to MC simulations of bone marrow *S*-values by Snyder et al. in MIRD Pamphlet No. 11 [15, 33]. Later, Cristy and Eckerman and Bouchet et al. refined these to model the role of photon absorbed fractions and calculate values for several skeletal regions according to the MIRD schema [34, 35]. Similarly, Hemmingsson et al. generated and compared *S*-values for ^177^Lu, ^90^Y, and ^161^ Tb for human BM from male and female image-based dosimetry models [36]. In mice, Hui et al. defined a model for marrow dose using ellipsoids, spheroids, and cylinders, computing SAFs of 15–20% for ^90^Y [37]. Muthuswamy et al. built on this model to estimate absorbed fractions for additional radionuclides, showing in particular that the cross-dose contribution is non-negligible compared to local beta energy deposition – illustrating the utility of organ-organ S-values in accurately estimating marrow dose [38]*.* Kostou et al., in the aforementioned study simulating heterogeneous whole-body sources, estimated *S*-values targeting the bone marrow for [^18^F]FDG, [^68^ Ga]Ga—RGD, Free ^131^I, [^111^In]In—DTPA, [^177^Lu]Lu—Tetulomab and [^99m^Tc]Tc—HMPAO [22]. Dosimetry studies have also simulated subcutaneous tumour xenografts to investigate the implications of radiopharmaceutical uptake in tumours for absorbed dose to nearby tissues (Table 1). Bitar et al. considered a 30 g female mouse model with a 100 mg tumour inoculated on the right flank, while Larsson et al. simulated *S*-values in the MOBY phantom for various radionuclides, tumour sizes, and tumour placements, demonstrating that xenografts located on the flanks may lead to different patterns of dose deposition compared to those on the thighs, due to variations in tissue density and proximity to BM-rich regions [23, 39, 40]. These factors underline the importance of considering xenograft placement in dose simulations. Most recently, Tamborino et al. simulated a dataset of organ-organ *S*-values in the MOBY phantom to estimate dose to BM within different subregions of the skeleton, as well as from tumour xenografts on the left lower flank of the mouse [41]. To the best of our knowledge, simulation of BM *S*-values in mice has not incorporated dose deposited from tumour xenografts in the shoulder, as is commonly encountered in experimental settings. Uptake in such xenografts could represent a significant contribution to marrow cross-dose given the presence of multiple large bones in this region, and the relatively small number of soft-tissue interfaces separating these bones from the radionuclide source. Addressing this gap could improve the accuracy of dose estimations in preclinical models. While BM toxicity is a concern in RPT, the contribution of tumour-derived cross-dose remains underexplored in preclinical models. In clinical settings, it has been observed that tumour uptake contributes non-negligibly to BM dose, with implications for hematopoietic toxicity that vary according to the tumour burden [42, 43]. Translating such findings to preclinical models is complex, as murine xenografts often exhibit different growth patterns and microenvironments compared to human cancers, which can alter BM dose deposition [23, 44–46].

The aim of this study was to determine *S*-values that would allow to have BM absorbed dose estimates for ^177^Lu therapies in preclinical studies with mice including tumor xenografts in the shoulder. By incorporating tumour burden into *S*-value calculations, this study provides a refined dosimetric framework to improve BM dose assessments and support preclinical-to-clinical translation. Different configurations of the MOBY mouse phantom including artificially placed tumors to mimic a xenograft mouse model were used to simulate the marrow self- and cross-doses. We hope this helps the field to better perform preclinical therapeutic studies.

## Methods

### Mouse phantom generation

**Fig. 1:**
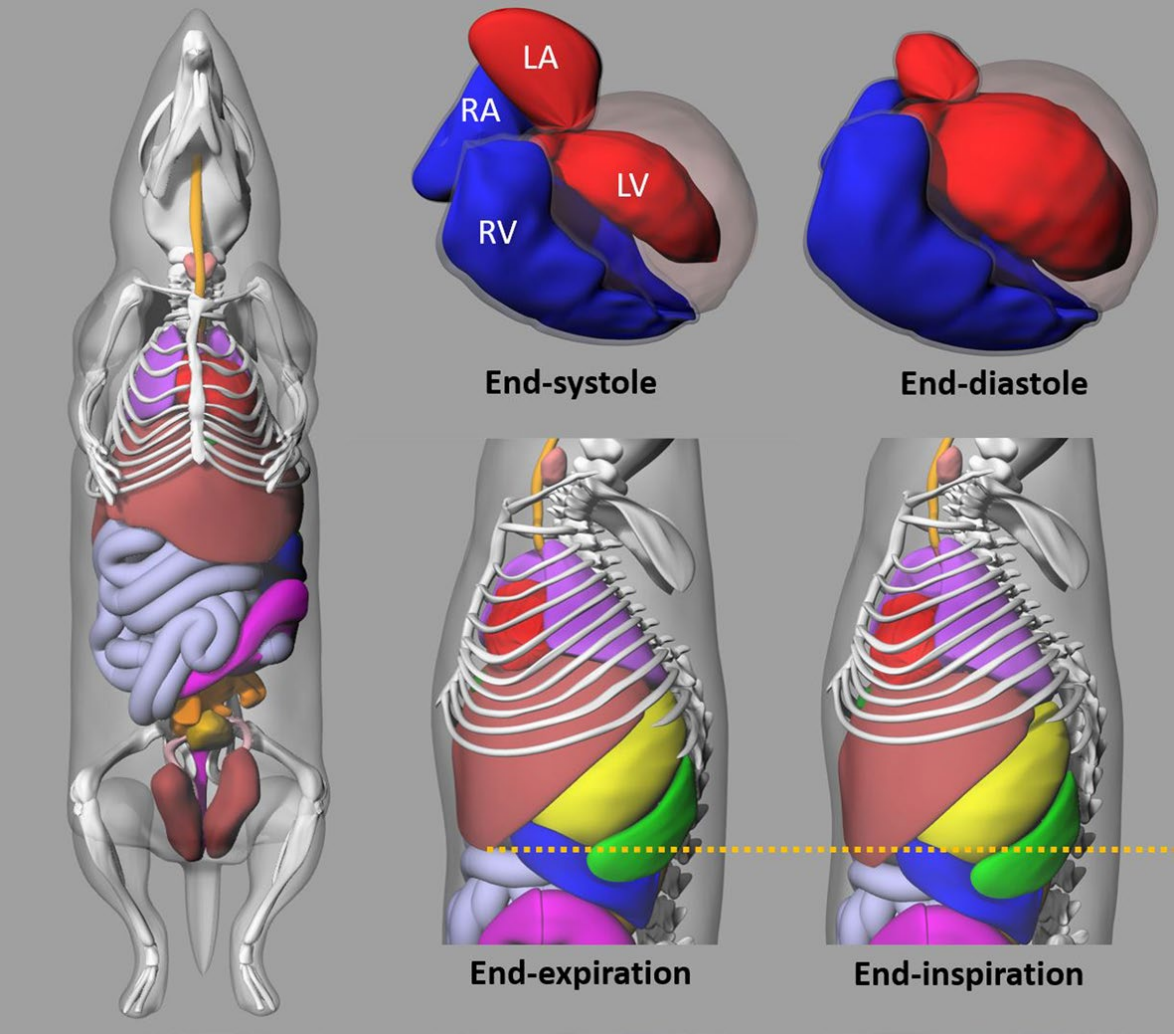
4D Mouse Whole Body (MOBY) computational phantom. The MOBY program allows for manipulation of physiological and anatomical parameters, including whole-body and individual organ size and respiratory and cardiac motion, in order to simulate the desired mouse model

The hybrid MOBY phantom, based on NURBS surfaces, allows for flexible segmentation or scaling of the mouse anatomy by user manipulation of the accompanying software package. The geometry of bones in the MOBY phantom was defined by segmenting the magnetic resonance microscopy (MRM) data and fitting NURBS surfaces to them in the Rhinoceros modelling software [17]. The BM cavity for each bone was defined by setting an inner shell within the surface at a uniform thickness, with the thickness determined based on typical measurements for each bone. Material compositions, consisting of material densities and elemental compositions, were based on human standards from ICRP Report 89 [17, 48, 49]. The MOBY phantom software package, which includes a user-controlled parameter file used as input to the program, allows for the creation of a 3D voxelized phantom with customized mouse anatomy, matrix and voxel dimensions, and lesion simulation options as desired (Fig. 1) [17]. Here, two mouse models, of different voxel resolutions and approximately equal mass (25 g), were generated using the MOBY program in Linux, the license for which was obtained from Segars at Duke University School of Medicine. The first resolution was set to a matrix size of 128 × 128x400 voxels with a cubic voxel size of (0.29 mm)^3^, the latter chosen to create enhanced resolution relative to both the mean range in tissue (~ 0.5 mm) of ^177^Lu beta emissions and the dimensions of BM in the majority of skeletal components throughout the body, as modelled by Muthuswamy [38, 50]. The second resolution – of 74 × 74x184 voxels and voxel size (0.625 mm)^3^ – was set such that the body mass and voxel size would match those of Keenan et al., whose simulations provided values for RADAR used here in the validation procedure [18]. These are hereafter referred to respectively as the ‘high resolution’ (HR) and ‘low-resolution’ (LR) phantoms.

In the generation of phantoms, the MOBY software outputs two 3D voxelized mouse images: a map of tissue attenuation values at a default radionuclide energy of 140 keV, and a time-integrated activity map for each tissue determined by user input in the parameter file. In this case, each tissue was assigned a unique time-integrated activity, creating a mask that was later used in MATLAB R2023a (Mathworks Inc.)  to generate the voxelized source images for the MC simulations. The full list of segmented tissues is provided in Supplemental Table A1. In particular, the brain and heart were each treated as uniform organs; the large and small intestines and the stomach were treated as hollow organs with no activity assigned to the cavities; and the components of the skeleton, including the bone marrow, were all separated in the activity map as allowed by the MOBY program. The whole body was scaled equally along each axis to achieve the desired mouse mass of 25 g; however, no scaling factors were applied to individual organs in the parameter file. Both LR and HR mice were produced in full exhale with the heart in end-diastole phase. The total BM mass in the phantom was 1.20 g and 0.73 g in the LR and HR configurations respectively, computed using a density of 1.03 g/cm^3^ from Woodard and White [51].

### Tumour generation

**Fig. 2:**
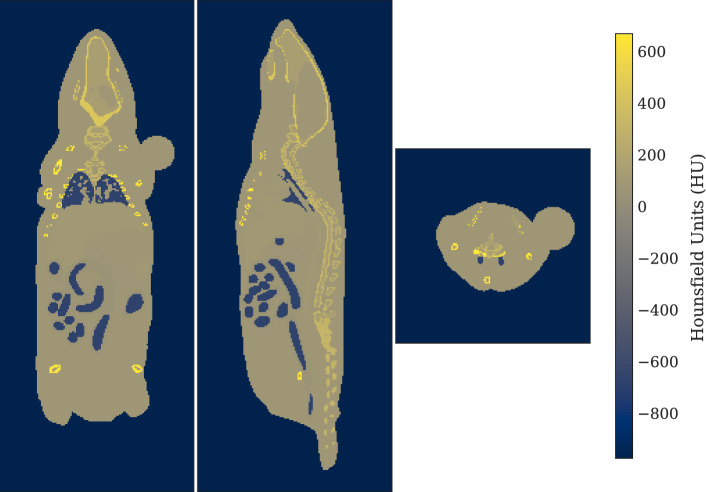
MOBY 25 g mouse phantom attenuation map with tumour xenograft in left shoulder. Phantom resolution: HR. Tumour mass: 0.54 g. Left: coronal view. Middle: sagittal view. Right: transverse view

To simulate dose deposition to the BM in the preclinical setting of xenograft tumor-bearing mice, spherical tumours were placed subcutaneously in the left shoulder of the mouse for additional Monte Carlo simulations. First, the MOBY program was used to generate separate activity and attenuation images for the tumour, placed using coordinates determined from inspection of live mouse PET-CT images. These images were then merged with the respective images for the mouse in MATLAB (Fig. 2), while the matrix sizes of the two phantoms were expanded with voxels of zero value to 80 × 80×175 and 140 × 140×355, at LR and HR respectively. In total, ten lesion sizes were simulated, ranging approximately 0.1–1.2 g, with the diameters – determined by assuming a lesion density of 1.0 g/cm^3^ – ranging 4.2–8.8 mm (equivalent to ~ 14–30 voxels) at HR and 4.3–9.0 mm (~ 7–14 voxels) at LR. The dimensions of the smallest tumour realisations were thus approximately eight times the mean range (~ 0.5 mm) and twice the maximum range (~ 2 mm) of ^177^Lu beta emissions in tissue, and hence the self-absorbed dose fraction was anticipated to predominate across all tumour simulations [50].

### GATE Monte Carlo simulations

GATE (Geant4 Application for Emission Tomography) is a Monte Carlo simulation platform, popular in radiotherapy and dosimetry applications [52–55]*.* Here, ^177^Lu dosimetry simulations were performed in GATE v9.3 inside of the MOBY phantom – in particular, the physics list emstandard_opt3 was used to simulate the electromagnetic interactions of the primary emissions within the tissues of the mouse. The ^177^Lu source was defined using the GATE in-built ion source command in the macro file. Range cut-offs for gammas, electrons and positrons were set to 0.001 mm within the phantom and 1 mm elsewhere in the simulation world.

The GATE program takes several inputs including multiple material database files, a 3D attenuation map – analogous to a CT image – and a 3D activity image locating the radionuclide source. In this case, a source image for each organ was generated in MATLAB as a normalized time-integrated activity image, by first using the mouse segmentation mask generated in MOBY to select the tissue, then placing a single unit of activity uniformly across all tissue voxels and normalizing by the number of voxels in the tissue, thus effectively giving a new voxelized mouse image with equal emission probability everywhere in the source tissue and zero everywhere else. The attenuation map image, generated by MOBY in units of 1/pixel, was converted to Hounsfield Units (HU) for input to GATE, by first dividing by the voxel size to yield units of 1/cm, then interpolating from the HU-to-µ conversion curve described by Schneider et al. [56].

GATE simulation results were computed and stored in 3D voxelized formats, using a *DoseActor* with dimensions equal to that of the phantom matrix – for this study, distributions of absorbed dose, squared dose, and dose uncertainty were of interest. For each source organ, a total of 5 × 10^9^ primaries (decays) were simulated via 40 parallelly executed simulations of 1.25 × 10^8^ primaries each, using the computing resources of the high-performance cluster Compute Canada [57].The split-simulation results were later merged (see *Dose analysis and computation of S-values*) to give a single dose and dose uncertainty distribution per source organ. This split-simulation procedure was repeated for all segmented organs in the phantom. The time required (mean ± SD) to complete MC simulations across all source tissues (including tumours) was 44.1 ± 0.4 h and 37.7 ± 0.3 h in the HR and LR phantoms respectively.

### Dose analysis and computation of S-values

The splitted simulations results were merged in MATLAB by taking a weighted average across the different dose distributions.

The statistical dose uncertainty of the merged simulations was estimated according to the history-by-history method as

$$\delta {D}_{k}=\sqrt{\frac{1}{N-1}\left(\frac{\sum_{i=1}^N{D}_{k,i}^{2}}{N}-{\left(\frac{\sum_{i=1}^N{D}_{k,i}}{N}\right)}^{2}\right)}$$

$${\varepsilon }_{{D}_{k}}=\frac{\delta {D}_{k}}{{D}_{k}}$$

where ${D}_{k}$ is the merged absorbed dose in Gy and $\delta {D}_{k}$ and ${\varepsilon }_{{D}_{k}}$ are, respectively, the statistical and relative dose uncertainties in voxel $k$ [58, 59].

To compute the dose deposited in the various organs of the mouse, organs of interest were segmented in the merged dose distribution according to their corresponding values in the activity map. Then, the *S*-value for a target organ, in Gy/decay (Gy Bq^−1^ s^−1^), was computed as

$$S\left({r}_{T}\leftarrow {r}_{S}\right)=\frac{\overline{D}\left({r}_{T}\leftarrow {r}_{S}\right)}{N}$$

where ${r}_{S}$ is the source organ for a given simulation, $N$ is the number of primaries (decays) simulated, and $\overline{D}\left( {r_{T} \leftarrow r_{s} } \right)$ is the mean dose in Gy in a target organ of $v$ voxels:

$$\overline{D}\left({r}_{T}\leftarrow {r}_{S}\right)=\frac{{\sum }^{v}{D}_{k}}{v}$$

Following error propagation principles, the *S*-value uncertainty $\delta S$ was computed as:

$$\delta S=\frac{\delta \overline{D}\left({r}_{T}\leftarrow {r}_{S}\right)}{N}=\frac{\sqrt{{\sum }^{v}\delta {D}_{k}^{2}}}{Nv}$$

The rationale of simulating 5 × 10^9^ primaries for each source organ was to achieve voxel-wise relative dose errors at or below 20% within the mouse phantom, in particular within tissues relevant in RPT contexts.

The effect of tumour size on dose to tissues of interest (in particular, certain significant skeletal regions) was investigated by computing the change in *S*-value relative to that simulated for the smallest tumour size:

$$\frac{S\left({r}_{T}\leftarrow Tumour, m\right)-S\left({r}_{T}\leftarrow Tumour, {m}_{0}\right)}{S\left({r}_{T}\leftarrow Tumour, {m}_{0}\right)}\times 100\%$$

where $m$ is a given tumour mass and ${m}_{0}$ is the mass of the smallest tumour at the given phantom resolution (0.12 g at HR and 0.13 g at LR).

### Validation of S-values

To validate and benchmark the GATE MC simulation methodology for computing *S*-values, reference values from the OLINDA v2.2.3 code (Hermes Medical Solutions) – also simulated in the MOBY phantom – were compared to those obtained in the current study. Before computing the arithmetic differences, *S*-values were scaled to the organ mass of the OLINDA phantom:

$${S}_{corrected}={S}_{computed}\frac{{m}_{MOBY}}{{m}_{OLINDA}}$$

OLINDA *S*-values for the 25 g mice were chosen for the proximity of the mouse and organ masses to those of this study (Table 2). Masses for this study’s phantoms, *m*_*MOBY*_ were computed using tissues densities from Woodard and White [51] and Boutaleb et al. [60]*.* Deviations between these masses and those from OLINDA may be due to small differences in phantom parameters, such as organ-specific or whole-body scaling factors, or those controlling respiratory and cardiac motion.

**Table 1:** Brief summary of selected mouse models for preclinical dosimetry

Model		Year	Type	Notes
Hui et al. [37]		1994	Mathematical, geometrical	Simplified geometrical shapes based on 25 g mice; femur BM cavity modelled as 0.1 cm × 0.9 cm cylinder
Muthuswamy et al. [38]		1998	Mathematical, geometrical	BM components modelled as slabs, cylinders or spheres depending on skeletal region
Flynn et al. [47]		2001	Mathematical	Built on Hui model; accounted for dose heterogeneity in kidneys and tumour; bones and BM modelled as cylinders
Bitar et al. [39, 40]		2007	Voxel-based nude mouse	Female 30 g nude mouse; 100 mg tumour inoculated on right flank
MOBY (Segars)	V1 [17]	2004	Computational phantom, voxelized	Based on 33 g mouse; defined using flexible NURBS surfaces fitted to 3D MRM data
	V2[48]	2009		Added heart motion; enhanced bone definition in skull, hand and foot regions

Relative differences in corrected *S*-values from OLINDA were then computed as:

$$\% difference=\frac{{S}_{corrected}-{S}_{OLINDA}}{{S}_{OLINDA}}\times 100\%$$

## Results

Absorbed dose was simulated by GATE in the MOBY phantom for all 26 source organs, from which *S*-values were computed for all 26 target tissues and an additional target volume representing the entire skeleton.

Computed *S*-values for both phantom resolutions are displayed in Tables 3 and 4, along with the corresponding uncertainties. LR phantom (0.625 mm voxel size) simulations yielded lower uncertainties than those in the HR phantom (0.29 mm), validating the inverse relationship between dose uncertainty and the number of primaries captured in each voxel. Uncertainties in *S* for all source-target combinations ranged 10^–13^–10^–9^%, while voxel-wise relative dose uncertainties within the mouse phantom ranged from below 1% in the source organ to ~ 20% in regions furthest away.

**Fig. 3:**
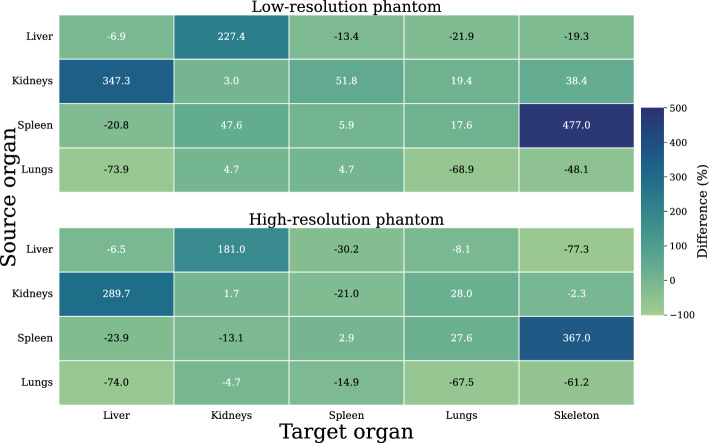
Comparison of S-values simulated in two MOBY mouse phantoms against OLINDA 25 g mouse reference values for select source-target organ combinations. Low-resolution (LR) phantom: 74 × 74×184 voxels, 0.625 mm cubic voxel size. High-resolution (HR) phantom: 128 × 128×400 voxels, 0.29 mm cubic voxel size

Results of *S*-value comparison to OLINDA for a selection of organs are displayed in Fig. 3 – these selected organs are of interest due to their prominent role in RPT. Self-dose *S*-values were typically within 2–7% of agreement with OLINDA for both phantom resolutions, except for the lungs. Cross-dose values were generally within 5–77% except for those between the kidneys and the liver and between the spleen and skeleton, whose OLINDA deviations were almost an order of magnitude larger than the rest despite having much lower mass deviations (4–8% for the liver, and 9–10% for the kidneys). Similar differences have been observed and explained in the literature [22, 24].

**Fig. 4:**
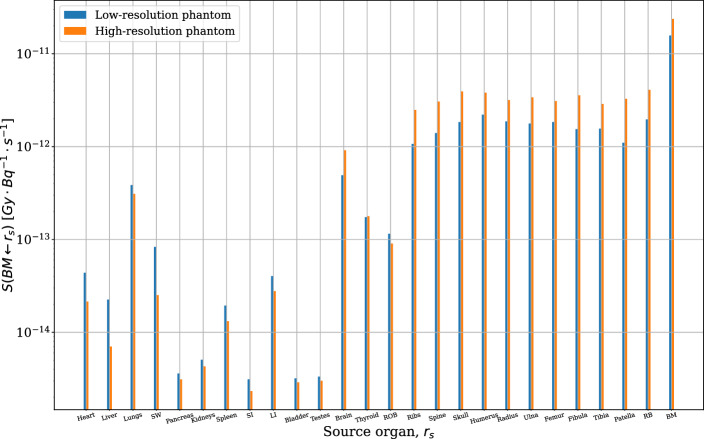
Organ *S*-values targeting the mouse bone marrow, for all simulated source organs. Low-resolution (LR) phantom: 74 × 74×184 voxels, 0.625 mm cubic voxel size. High-resolution (HR) phantom: 128 × 128×400 voxels, 0.29 mm cubic voxel size

Newly simulated BM S-values showed the largest absorbed dose per unit decay was due to the BM marrow self-irradiation followed by cross dose deposition from the considered skeletal regions (Fig. 4). Generally, high-resolution BM dose factors are greater than those simulated at low resolution for source tissues in the skeleton and near it (brain and thyroid), and vice versa for the remaining tissues. Additionally, for this subset of S-values, uncertainty inversely correlates with *S*-value magnitude. Select *S*-values for the BM as target were also compared to those from Tamborino et al., who simulated a MOBY phantom of comparable voxel resolution (0.234 mm) but differing tissue composition to the phantoms used here; the values in this paper underestimate theirs by 6–87%, varying with the size of the source organ and the voxel resolution of the compared phantom. Differences were generally lower for the low resolution (0.625 mm) phantom, as well as for smaller source organs, such as the kidneys (Table 6).

For almost all targets, estimated *S*-value dose contributions from tumours decrease with tumour size, including for the self-dose (Table 5, Fig. 6), while uncertainties are relatively constant and similarly negligible as those for the organ simulations. Relative to the smallest tumours, self-dose *S*-values decrease by ~ 80%, while cross-doses to the majority of significant skeletal components, including BM, decrease by up to 20%. The components excepted from this trend are the femur and tibia, whose S-values either increase over the range of tumour sizes or decrease by less than 5% (Fig. 6).

## Discussion

In this work, we performed MC simulation to obtain *S*-values for BM dosimetry for preclinical application in dosimetry during radiopharmaceutical development. MC simulations were performed for different resolutions of the MOBY mouse phantom and results were validated against previously published *S*-values for different source-target combinations.

The validation procedure showed the computed *S*-values were of variable accuracy relative to previously published OLINDA data. First, it should be noted that, when creating the two phantoms used in this study, only voxel size and whole-body size were varied in the MOBY program, where other studies may have varied other parameters such as organ volumes to match the masses and geometries of their reference phantoms more closely. Thus, matching the voxel size or total body mass of the LR phantom to that of the OLINDA reference phantom did not guarantee similarly equal organ geometry; in many cases, organ masses discrepancies were greater than 50%, approaching 65% for the heart and 137% for testes. In other cases, LR organ mass differences were not as large as above, but were still greater than those for the HR phantom whose voxel size did not match OLINDA; the skeleton mass, for instance, was 52% greater at LR, versus 15% at HR (Table 2). *S*-value agreement was similarly unpredictable: while tissues with similar masses produced similar self-dose values, tissue mass agreement did not guarantee agreement of cross-dose factors, particularly for those between the kidneys and liver (Fig. 3). Notably, Kostou et al., when validating against Keenan, found similarly anomalous large deviations (20–32%) for kidney-liver *S*-values, despite designing their phantom to match Keenan’s as closely as possible in organ masses [22]. This indicates that while self- and cross-doses are determined by factors such as phantom type (voxelized or analytical), voxel size and resolution, and mouse and organ masses, cross-doses also vary with other factors like organ geometry and mouse anatomy, determining the relative positioning of organs [22, 61]. Even when attempting to control for such factors, *S*-values may still deviate from those computed in reference models like OLINDA [24]. Generally, large deviations are observed for tissues that are small relative to the voxel size or exhibit complex anatomical distributions—such as the lungs, kidneys, and skeleton—where even minor differences in tissue mass can significantly alter radionuclide energy deposition patterns. These discrepancies are particularly pronounced in organs with dimensions comparable to the range of ^1^⁷⁷Lu beta emissions. For example, the kidneys and lungs are much smaller than the liver, yet their characteristic dimensions are on the order of a few voxel lengths in the LR phantom, making dose calculations highly sensitive to voxel discretization. Furthermore, the limited thickness of some lung regions results in a greater fraction of cross-dose contributions from surrounding tissues compared to larger, more compact organs like the liver. Similarly, the skeleton, composed of elongated and thin structures, presents additional challenges due to its intricate geometry, which may not be well resolved at lower voxel resolutions. Lung composition also introduces further variability due to its lower density and heterogeneous microstructure [62–64]. These anatomical and dosimetric factors must be considered when comparing S-values across different models, particularly in the context of translating preclinical dosimetry to clinical applications [65]. It is particularly important to ensure consistency in self-dose factors, which are the predominant contributors to absorbed dose and can differ by up to 70% even in mouse models with similar body masses. This is also demonstrated in the later comparison of BM *S*-values to those from Tamborino et al., where all differences were in the range 6–87%. These deviations are attributable to several factors, including differences in mouse anatomy and organ masses – BM mass differed by -26% and -55% for our LR and HR phantoms respectively; differing tissue compositions of the two mouse models – Tamborino considered the skeleton to be composed exclusively of soft tissue with density 1.0 g/cm^3^ to allow for rescaling of each skeletal region by marrow cellularity; and the selection of decay database used to define the radionuclide in the simulations.

**Table 2:** Organ masses of the OLINDA 25 g mouse and the MOBY phantoms of this study

Organ	OLINDA 25 g mouse (g)	LR phantom (g)	Difference (%)	HR phantom (g)	Difference (%)
Heart	0.235	0.388	*65.0*	0.308	*31.1*
Liver	1.74	1.87	*7.89*	1.80	*3.50*
Lungs	0.087	0.115	*32.6*	0.124	*42.0*
Stomach wall	0.055	0.066	*19.1*	0.068	*22.2*
Pancreas	0.305	0.371	*21.7*	0.347	*13.7*
Kidneys	0.302	0.364	*20.5*	0.330	*9.46*
Spleen	0.111	0.134	*20.5*	0.107	*−3.40*
Small intestine	1.74	0.998	−*42.7*	0.984	*−43.5*
Large intestine	0.583	0.308	*−47.3*	0.308	*−47.1*
Bladder	0.060	0.071	*18.7*	0.064	*6.66*
Testes	0.160	0.380	*137*	0.324	*102*
Brain	0.466	0.559	*20.1*	0.489	*5.01*
Thyroid	0.014	0.015	*7.95*	0.013	*−9.59*
Skeleton	2.18	3.33	*52.6*	2.51	*15.0*
Total body	24.1	26.2	*8.57*	25.2	*4.46*

^*^Low-resolution (LR) phantom: 74 × 74x184 voxels, 0.625 mm voxel size

High-resolution (HR) phantom: 128 × 128x400 voxels, 0.29 mm voxel size

The use of italics is to distinguish the columns with 'relative difference' data from those with measures of S-values or organ masses

Varying MOBY phantom voxel resolution and matrix size in MC simulations has the potential to influence the nature and frequency of radionuclide interactions in each voxel, which in turn affects the resulting estimated absorbed doses and dose uncertainties. While a decrease in resolution did not significantly increase accuracy against OLINDA (Fig. 3), it had the effect of reducing dose and* S*-value uncertainties across all simulations (Tables 3 and 4). This was expected, since increasing voxel size was hypothesized to increase the number of primaries captured per voxel, which in turn scales inversely with uncertainty. Despite decreased uncertainties, *S*-values computed at LR are not necessarily superior, as a smaller voxel size may be preferable for modeling radionuclide interactions in small tissues, particularly with regard to BM. For instance, Kostou et al. decreased voxel size to 0.5 mm from 0.625 mm when simulating BM *S*-values from whole-body source distributions [22]. Similarly, Tamborino et al. used a voxel size of 0.234 mm in their simulation of organ S-values, which included BM [41]. While larger voxel sizes are capable of reducing statistical uncertainties in MC simulations, they may overestimate the bone-BM interface surface area, leading to cross-dose errors of up to 25% for low-energy beta emitters [66]. For *β*-particle dosimetry, voxel sizes close to or smaller than the mean range of emissions (~ 0.5 mm for ^177^Lu) provide more accurate absorbed dose distributions, particularly in heterogeneous tissues like the skeleton [38]. However, further research is needed to determine the optimal balance between voxel resolution and computational efficiency, particularly for preclinical models, where anatomical fidelity is crucial for clinical translation [65, 67].

**Table 3:** *S*-values, low resolution (LR) phantom

	S [Gy/Bq⋅s] (Relative uncertainty [%])							
	Target organ							
	Heart	Liver	Lungs	Stomach wall	Pancreas	Kidneys	Spleen	Small intestine
	*Source organ*							
Heart	5.29E-11 (2.95E-13)	2.56E-13 (1.94E-12)	9.30E-13 (2.25E-12)	8.72E-14 (1.75E-11)	1.93E-14 (1.45E-11)	1.16E-13 (6.57E-12)	5.71E-15 (3.08E-11)	2.24E-14 (8.92E-12)
Liver	2.57E-13 (4.22E-12)	1.15E-11 (2.88E-13)	3.57E-13 (4.86E-12)	5.05E-13 (7.45E-12)	1.96E-13 (4.98E-12)	1.30E-13 (6.20E-12)	6.89E-15 (2.60E-11)	8.61E-14 (4.52E-12)
Lungs	9.30E-13 (2.31E-12)	1.52E-13 (2.49E-12)	4.88E-11 (3.08E-13)	9.87E-14 (1.65E-11)	4.96E-15 (2.52E-11)	4.10E-15 (2.17E-11)	4.26E-15 (3.55E-11)	2.90E-15 (2.08E-11)
Stomach wall	8.70E-14 (7.22E-12)	6.10E-13 (1.35E-12)	9.87E-14 (6.73E-12)	2.40E-10 (3.50E-13)	4.37E-12 (1.31E-12)	8.94E-14 (7.32E-12)	1.37E-12 (3.22E-12)	1.48E-13 (4.47E-12)
Pancreas	1.92E-14 (1.30E-11)	6.83E-13 (1.51E-12)	4.90E-15 (1.81E-11)	4.37E-12 (3.06E-12)	9.32E-11 (2.83E-13)	9.22E-13 (2.54E-12)	4.34E-12 (2.31E-12)	1.24E-12 (1.56E-12)
Kidneys	1.16E-13 (6.34E-12)	2.09E-13 (2.51E-12)	4.06E-15 (1.99E-11)	8.93E-14 (1.71E-11)	9.22E-13 (2.58E-12)	6.42E-11 (2.93E-13)	2.57E-13 (7.32E-12)	3.89E-13 (2.83E-12)
Spleen	5.68E-15 (1.69E-11)	7.05E-15 (7.72E-12)	4.24E-15 (1.95E-11)	1.37E-12 (4.51E-12)	4.34E-12 (1.38E-12)	2.57E-13 (4.41E-12)	1.77E-10 (2.97E-13)	6.01E-15 (1.43E-11)
Small intestine	2.23E-14 (1.38E-11)	2.80E-13 (2.38E-12)	2.88E-15 (2.37E-11)	1.48E-13 (1.68E-11)	1.24E-12 (2.53E-12)	3.89E-13 (4.65E-12)	5.99E-15 (3.17E-11)	2.88E-11 (3.10E-13)
Large intestine	4.27E-15 (1.96E-11)	6.28E-14 (4.71E-12)	2.14E-15 (2.74E-11)	1.60E-14 (4.49E-11)	2.67E-12 (1.75E-12)	7.49E-13 (2.95E-12)	9.53E-14 (1.59E-11)	1.38E-12 (1.45E-12)
Bladder	1.44E-15 (3.35E-11)	1.84E-15 (1.59E-11)	9.75E-16 (4.05E-11)	2.07E-15 (7.42E-11)	3.40E-15 (3.07E-11)	3.63E-15 (2.34E-11)	2.54E-15 (4.84E-11)	8.91E-15 (1.11E-11)
Testes	8.05E-16 (4.48E-11)	1.02E-15 (2.13E-11)	5.94E-16 (5.21E-11)	1.12E-15 (1.00E-10)	1.68E-15 (4.36E-11)	1.84E-15 (3.29E-11)	1.38E-15 (6.52E-11)	3.36E-15 (1.83E-11)
Brain	2.77E-15 (2.42E-11)	1.55E-15 (1.64E-11)	3.52E-15 (2.19E-11)	1.36E-15 (8.87E-11)	9.42E-16 (5.78E-11)	8.58E-16 (4.78E-11)	9.67E-16 (7.55E-11)	6.62E-16 (4.35E-11)
Thyroid	7.73E-15 (1.45E-11)	3.03E-15 (1.17E-11)	9.45E-15 (1.35E-11)	2.22E-15 (6.96E-11)	1.58E-15 (4.48E-11)	1.32E-15 (3.84E-11)	1.48E-15 (6.10E-11)	1.09E-15 (3.40E-11)
ROB	6.05E-14 (8.66E-12)	3.33E-14 (5.22E-12)	1.40E-13 (7.75E-12)	1.29E-13 (1.47E-11)	3.87E-14 (1.11E-11)	5.85E-14 (9.20E-12)	6.22E-14 (1.46E-11)	1.03E-13 (4.21E-12)
Ribs	2.17E-13 (4.59E-12)	3.34E-13 (1.87E-12)	1.42E-12 (1.90E-12)	5.10E-13 (7.39E-12)	6.65E-15 (2.20E-11)	5.15E-15 (1.94E-11)	4.86E-14 (1.66E-11)	4.19E-15 (1.76E-11)
Spine	4.26E-14 (1.01E-11)	3.73E-15 (1.06E-11)	3.06E-13 (4.12E-12)	1.07E-14 (4.43E-11)	3.91E-15 (2.77E-11)	6.50E-15 (1.73E-11)	4.16E-15 (3.63E-11)	3.94E-15 (1.72E-11)
Skull	2.27E-15 (2.67E-11)	1.29E-15 (1.80E-11)	2.83E-15 (2.45E-11)	1.10E-15 (9.89E-11)	7.95E-16 (6.33E-11)	7.19E-16 (5.24E-11)	7.97E-16 (8.33E-11)	5.70E-16 (4.71E-11)
Humerus	1.02E-14 (1.26E-11)	4.83E-15 (9.26E-12)	1.72E-14 (1.12E-11)	3.45E-15 (5.58E-11)	2.34E-15 (3.69E-11)	1.97E-15 (3.16E-11)	2.16E-15 (5.03E-11)	1.54E-15 (2.86E-11)
Radius	1.14E-14 (1.19E-11)	9.74E-15 (6.58E-12)	1.60E-14 (1.00E-11)	6.16E-15 (4.20E-11)	4.29E-15 (2.74E-11)	3.27E-15 (2.45E-11)	3.58E-15 (3.94E-11)	2.78E-15 (2.14E-11)
Ulna	1.07E-14 (1.23E-11)	8.88E-15 (6.17E-12)	1.17E-14 (2.07E-11)	5.62E-15 (4.17E-11)	4.10E-15 (2.07E-11)	2.92E-15 (2.49E-11)	3.26E-15 (3.81E-11)	2.64E-15 (1.57E-11)
Femur	9.71E-16 (4.08E-11)	1.22E-15 (1.95E-11)	7.11E-16 (4.77E-11)	1.37E-15 (9.09E-11)	2.08E-15 (3.91E-11)	2.43E-15 (2.88E-11)	1.78E-15 (5.74E-11)	4.12E-15 (1.64E-11)
Fibula	5.71E-16 (5.34E-11)	7.18E-16 (2.16E-11)	4.51E-16 (1.06E-10)	7.89E-16 (1.12E-10)	1.13E-15 (3.93E-11)	1.23E-15 (3.83E-11)	9.38E-16 (7.09E-11)	1.95E-15 (1.82E-11)
Tibia	4.95E-16 (5.74E-11)	6.31E-16 (2.32E-11)	4.01E-16 (1.12E-10)	6.70E-16 (1.21E-10)	9.73E-16 (4.26E-11)	1.04E-15 (4.17E-11)	7.98E-16 (7.70E-11)	1.68E-15 (1.97E-11)
Patella	6.32E-16 (5.08E-11)	8.19E-16 (2.03E-11)	5.07E-16 (9.99E-11)	8.95E-16 (1.05E-10)	1.30E-15 (3.68E-11)	1.36E-15 (3.66E-11)	1.04E-15 (6.74E-11)	2.35E-15 (1.67E-11)
Remaining bones	8.27E-14 (7.45E-12)	8.45E-15 (8.96E-12)	3.61E-14 (1.49E-11)	2.82E-15 (5.88E-11)	3.12E-15 (2.36E-11)	3.48E-15 (2.27E-11)	2.50E-15 (4.33E-11)	3.85E-15 (1.30E-11)
BM	4.35E-14 (1.00E-11)	2.34E-14 (6.31E-12)	3.84E-13 (3.71E-12)	8.30E-14 (1.82E-11)	3.39E-15 (3.01E-11)	4.65E-15 (2.05E-11)	1.91E-14 (2.59E-11)	2.89E-15 (2.04E-11)

	S [Gy/Bq⋅s] (Relative uncertainty [%])							
	Target organ							
	Large intestine	Bladder	Testes	Brain	Thyroid	ROB	Ribs	Spine
	*Source organ*							
Heart	4.31E-15 (2.81E-11)	1.45E-15 (7.81E-11)	8.06E-16 (5.66E-11)	2.89E-15 (1.99E-11)	8.16E-15 (8.61E-11)	5.85E-14 (1.30E-12)	2.17E-13 (6.03E-12)	4.30E-14 (9.67E-12)
Liver	3.34E-14 (1.28E-11)	1.83E-15 (6.97E-11)	1.01E-15 (4.10E-11)	1.51E-15 (2.77E-11)	3.02E-15 (1.18E-10)	3.36E-14 (1.70E-12)	2.36E-13 (5.22E-12)	4.61E-15 (1.77E-11)
Lungs	2.17E-15 (3.92E-11)	9.86E-16 (9.45E-11)	6.00E-16 (6.58E-11)	3.66E-15 (1.76E-11)	1.00E-14 (7.75E-11)	6.88E-14 (1.31E-12)	1.42E-12 (2.61E-12)	3.07E-13 (4.15E-12)
Stomach wall	1.61E-14 (2.11E-11)	2.08E-15 (6.53E-11)	1.12E-15 (4.89E-11)	1.39E-15 (2.86E-11)	2.36E-15 (1.61E-10)	1.35E-13 (9.03E-13)	5.10E-13 (4.04E-12)	1.11E-14 (1.71E-11)
Pancreas	2.67E-12 (1.93E-12)	3.39E-15 (5.13E-11)	1.67E-15 (3.82E-11)	9.67E-16 (3.44E-11)	1.67E-15 (1.88E-10)	1.07E-13 (1.21E-12)	6.56E-15 (2.09E-11)	4.24E-15 (1.94E-11)
Kidneys	7.49E-13 (3.42E-12)	3.61E-15 (4.96E-11)	1.83E-15 (3.68E-11)	8.81E-16 (3.60E-11)	1.40E-15 (2.09E-10)	6.70E-14 (1.29E-12)	5.11E-15 (2.38E-11)	7.12E-15 (1.47E-11)
Spleen	9.53E-14 (1.04E-11)	2.53E-15 (5.94E-11)	1.37E-15 (4.34E-11)	9.87E-16 (3.40E-11)	1.55E-15 (1.98E-10)	6.91E-14 (1.22E-12)	4.85E-14 (1.27E-11)	4.53E-15 (1.87E-11)
Small intestine	1.38E-12 (2.57E-12)	8.90E-15 (3.17E-11)	3.34E-15 (2.75E-11)	6.77E-16 (4.11E-11)	1.15E-15 (2.27E-10)	2.44E-13 (7.97E-13)	4.15E-15 (2.67E-11)	4.28E-15 (1.91E-11)
Large intestine	7.41E-11 (3.30E-13)	1.42E-14 (2.51E-11)	6.45E-15 (2.80E-11)	5.73E-16 (4.47E-11)	9.37E-16 (2.55E-10)	2.59E-13 (7.35E-13)	2.81E-15 (3.20E-11)	7.06E-15 (1.48E-11)
Bladder	1.43E-14 (1.45E-11)	2.90E-10 (2.94E-13)	1.02E-13 (6.49E-12)	3.48E-16 (5.74E-11)	5.32E-16 (3.39E-10)	1.27E-13 (8.98E-13)	1.26E-15 (4.78E-11)	4.92E-15 (1.75E-11)
Testes	6.47E-15 (2.24E-11)	1.02E-13 (1.47E-11)	7.24E-11 (6.26E-13)	2.34E-16 (7.02E-11)	3.36E-16 (4.13E-10)	3.70E-13 (1.53E-12)	7.37E-16 (6.25E-11)	8.97E-15 (1.75E-11)
Brain	5.56E-16 (7.78E-11)	3.36E-16 (1.63E-10)	2.26E-16 (9.84E-11)	3.90E-11 (2.87E-13)	1.70E-14 (5.92E-11)	1.12E-14 (2.68E-12)	2.30E-15 (3.68E-11)	1.18E-14 (1.77E-11)
Thyroid	8.76E-16 (6.15E-11)	4.96E-16 (1.34E-10)	3.27E-16 (9.29E-11)	1.66E-14 (8.33E-12)	1.58E-09 (3.11E-13)	3.18E-13 (6.89E-13)	6.05E-15 (2.29E-11)	2.49E-12 (1.63E-12)
ROB	1.52E-13 (6.26E-12)	1.27E-13 (1.41E-11)	9.31E-14 (7.16E-12)	1.07E-14 (1.54E-11)	1.61E-13 (2.75E-11)	1.23E-12 (2.87E-13)	2.25E-13 (5.49E-12)	1.56E-13 (5.04E-12)
Ribs	2.84E-15 (3.44E-11)	1.26E-15 (8.37E-11)	7.42E-16 (6.02E-11)	2.39E-15 (2.18E-11)	6.35E-15 (9.70E-11)	2.97E-13 (6.19E-13)	7.05E-11 (3.40E-13)	1.22E-13 (6.38E-12)
Spine	6.51E-15 (2.32E-11)	4.48E-15 (4.46E-11)	8.12E-15 (4.41E-11)	1.17E-14 (1.51E-11)	2.48E-12 (8.70E-12)	1.94E-13 (8.14E-13)	1.22E-13 (8.64E-12)	4.46E-11 (3.18E-13)
Skull	4.80E-16 (8.34E-11)	2.97E-16 (1.74E-10)	2.04E-16 (1.20E-10)	1.73E-12 (1.39E-12)	1.33E-14 (6.71E-11)	1.87E-13 (7.46E-13)	1.90E-15 (4.03E-11)	1.00E-13 (6.65E-12)
Humerus	1.21E-15 (5.25E-11)	6.46E-16 (1.17E-10)	4.13E-16 (7.71E-11)	6.32E-15 (1.35E-11)	2.02E-14 (5.64E-11)	3.84E-13 (6.02E-13)	1.29E-14 (2.08E-11)	7.52E-15 (2.01E-11)
Radius	2.05E-15 (4.05E-11)	9.99E-16 (9.46E-11)	6.19E-16 (7.75E-11)	2.56E-15 (2.11E-11)	5.27E-15 (1.07E-10)	2.27E-13 (6.81E-13)	1.74E-14 (1.35E-11)	3.12E-15 (2.49E-11)
Ulna	1.94E-15 (3.28E-11)	9.67E-16 (9.61E-11)	5.92E-16 (5.38E-11)	2.01E-15 (2.41E-11)	4.23E-15 (1.00E-10)	1.98E-13 (7.30E-13)	1.83E-14 (1.15E-11)	2.89E-15 (2.23E-11)
Femur	5.96E-15 (2.37E-11)	1.09E-14 (2.87E-11)	1.18E-14 (1.46E-11)	2.67E-16 (6.59E-11)	3.90E-16 (4.00E-10)	2.15E-13 (6.92E-13)	8.75E-16 (5.74E-11)	5.88E-15 (1.60E-11)
Fibula	2.90E-15 (2.69E-11)	6.03E-15 (3.87E-11)	1.49E-14 (1.07E-11)	1.77E-16 (8.19E-11)	2.53E-16 (4.12E-10)	2.72E-13 (6.24E-13)	7.03E-16 (5.92E-11)	3.41E-15 (2.06E-11)
Tibia	2.32E-15 (3.01E-11)	4.89E-15 (4.31E-11)	1.14E-14 (1.26E-11)	1.58E-16 (8.71E-11)	2.21E-16 (4.42E-10)	1.65E-13 (8.00E-13)	6.17E-16 (6.34E-11)	2.81E-15 (2.27E-11)
Patella	2.76E-15 (2.77E-11)	6.09E-15 (3.86E-11)	8.70E-15 (1.41E-11)	1.92E-16 (7.87E-11)	2.76E-16 (3.98E-10)	1.78E-13 (7.66E-13)	8.11E-16 (5.55E-11)	2.30E-15 (2.52E-11)
Remaining bones	1.18E-13 (7.12E-12)	5.75E-15 (3.95E-11)	5.42E-15 (1.79E-11)	2.59E-15 (2.12E-11)	8.52E-15 (7.05E-11)	1.97E-13 (7.30E-13)	1.19E-13 (7.45E-12)	8.61E-14 (6.60E-12)
BM	3.99E-14 (1.25E-11)	2.94E-15 (5.53E-11)	3.10E-15 (4.01E-11)	4.93E-13 (2.59E-12)	1.75E-13 (3.27E-11)	1.38E-13 (1.24E-12)	1.07E-12 (2.83E-12)	1.40E-12 (1.94E-12)

	S [Gy/Bq⋅s] (Relative uncertainty [%])							
	Target organ							
	Skull	Humerus	Radius	Ulna	Femur	Fibula	Tibia	Patella
	*Source organ*							
Heart	2.35E-15 (3.32E-11)	1.09E-14 (4.58E-11)	1.24E-14 (4.63E-11)	1.14E-14 (5.29E-11)	1.01E-15 (8.75E-11)	6.48E-16 (3.20E-10)	6.06E-16 (1.09E-10)	8.12E-16 (9.15E-10)
Liver	1.55E-15 (3.92E-11)	6.49E-15 (5.13E-11)	1.31E-14 (4.23E-11)	1.21E-14 (4.34E-11)	1.60E-15 (6.40E-11)	9.65E-16 (2.49E-10)	8.37E-16 (8.92E-11)	1.08E-15 (7.04E-10)
Lungs	2.93E-15 (2.98E-11)	1.88E-14 (3.87E-11)	1.74E-14 (3.89E-11)	1.23E-14 (4.80E-11)	7.38E-16 (1.02E-10)	5.08E-16 (3.62E-10)	4.74E-16 (1.24E-10)	6.18E-16 (9.77E-10)
Stomach wall	1.14E-15 (4.79E-11)	3.65E-15 (7.84E-11)	6.70E-15 (6.32E-11)	6.01E-15 (6.91E-11)	1.40E-15 (7.42E-11)	8.96E-16 (2.73E-10)	8.07E-16 (9.51E-11)	1.06E-15 (7.82E-10)
Pancreas	8.14E-16 (5.67E-11)	2.49E-15 (9.57E-11)	4.63E-15 (7.59E-11)	4.38E-15 (8.23E-11)	2.13E-15 (6.03E-11)	1.25E-15 (2.32E-10)	1.15E-15 (7.98E-11)	1.48E-15 (6.44E-10)
Kidneys	7.31E-16 (5.97E-11)	2.11E-15 (1.05E-10)	3.53E-15 (8.69E-11)	3.10E-15 (1.15E-10)	2.50E-15 (5.58E-11)	1.36E-15 (2.24E-10)	1.24E-15 (7.67E-11)	1.56E-15 (6.22E-10)
Spleen	8.18E-16 (5.66E-11)	2.27E-15 (9.97E-11)	3.86E-15 (8.33E-11)	3.53E-15 (9.87E-11)	1.81E-15 (6.53E-11)	1.11E-15 (2.47E-10)	9.56E-16 (8.75E-11)	1.23E-15 (7.08E-10)
Small intestine	5.81E-16 (6.72E-11)	1.62E-15 (1.19E-10)	2.99E-15 (9.44E-11)	2.82E-15 (1.04E-10)	4.20E-15 (4.30E-11)	2.14E-15 (1.79E-10)	1.97E-15 (6.09E-11)	2.65E-15 (4.69E-10)
Large intestine	4.90E-16 (7.30E-11)	1.28E-15 (1.33E-10)	2.20E-15 (1.10E-10)	2.13E-15 (1.19E-10)	6.01E-15 (3.60E-11)	3.22E-15 (1.45E-10)	2.77E-15 (5.12E-11)	3.24E-15 (4.38E-10)
Bladder	3.01E-16 (9.33E-11)	6.89E-16 (1.84E-10)	1.06E-15 (1.58E-10)	1.05E-15 (1.74E-10)	1.12E-14 (2.64E-11)	6.73E-15 (9.97E-11)	5.77E-15 (3.55E-11)	6.94E-15 (3.01E-10)
Testes	2.06E-16 (1.13E-10)	4.43E-16 (2.31E-10)	6.53E-16 (2.02E-10)	6.45E-16 (2.93E-10)	1.21E-14 (2.53E-11)	1.67E-14 (6.31E-11)	1.67E-14 (2.33E-11)	9.82E-15 (2.52E-10)
Brain	1.73E-12 (2.10E-12)	6.45E-15 (6.14E-11)	2.67E-15 (1.00E-10)	2.21E-15 (1.18E-10)	2.69E-16 (1.71E-10)	2.00E-16 (5.94E-10)	1.92E-16 (1.96E-10)	2.43E-16 (1.64E-09)
Thyroid	1.30E-14 (1.42E-11)	2.02E-14 (3.63E-11)	5.44E-15 (7.00E-11)	4.61E-15 (8.02E-11)	3.85E-16 (1.42E-10)	2.86E-16 (4.90E-10)	2.70E-16 (1.66E-10)	3.17E-16 (1.40E-09)
ROB	1.65E-13 (6.33E-12)	2.06E-13 (1.51E-11)	1.99E-13 (1.81E-11)	1.91E-13 (1.82E-11)	1.78E-13 (1.00E-11)	2.62E-13 (2.51E-11)	1.59E-13 (1.07E-11)	1.71E-13 (9.32E-11)
Ribs	1.96E-15 (3.65E-11)	1.37E-14 (5.26E-11)	1.90E-14 (3.89E-11)	1.45E-14 (4.79E-11)	8.98E-16 (9.27E-11)	6.24E-16 (3.32E-10)	5.74E-16 (1.13E-10)	7.28E-16 (9.37E-10)
Spine	9.99E-14 (8.63E-12)	7.65E-15 (7.60E-11)	3.21E-15 (9.15E-11)	2.53E-15 (1.08E-10)	5.43E-15 (3.79E-11)	3.21E-15 (1.45E-10)	2.82E-15 (5.05E-11)	2.15E-15 (5.25E-10)
Skull	6.64E-11 (3.30E-13)	5.18E-15 (6.82E-11)	2.16E-15 (1.11E-10)	1.83E-15 (1.25E-10)	2.34E-16 (1.83E-10)	1.79E-16 (6.24E-10)	1.71E-16 (2.08E-10)	2.35E-16 (1.67E-09)
Humerus	5.06E-15 (2.28E-11)	5.37E-10 (3.42E-13)	1.50E-12 (7.27E-12)	1.81E-12 (6.42E-12)	4.99E-16 (1.25E-10)	3.68E-16 (4.31E-10)	3.35E-16 (1.48E-10)	4.00E-16 (1.24E-09)
Radius	2.08E-15 (3.55E-11)	1.46E-12 (5.93E-12)	6.52E-10 (3.37E-13)	1.93E-11 (2.02E-12)	7.38E-16 (1.02E-10)	5.17E-16 (3.62E-10)	4.77E-16 (1.24E-10)	5.95E-16 (1.01E-09)
Ulna	2.07E-15 (3.40E-11)	1.35E-12 (6.00E-12)	1.35E-11 (2.23E-12)	5.39E-10 (3.39E-13)	9.14E-16 (8.54E-11)	5.92E-16 (3.20E-10)	5.25E-16 (1.14E-10)	9.93E-16 (7.63E-10)
Femur	2.35E-16 (1.06E-10)	5.14E-16 (2.14E-10)	7.89E-16 (1.84E-10)	7.40E-16 (1.92E-10)	2.02E-10 (3.26E-13)	1.38E-14 (6.97E-11)	1.71E-13 (1.10E-11)	7.94E-14 (1.18E-10)
Fibula	1.84E-16 (1.15E-10)	4.10E-16 (2.07E-10)	5.84E-16 (2.03E-10)	5.78E-16 (2.01E-10)	1.63E-14 (2.00E-11)	1.37E-09 (3.42E-13)	9.21E-13 (4.39E-12)	2.90E-14 (1.36E-10)
Tibia	1.65E-16 (1.22E-10)	3.60E-16 (2.20E-10)	5.31E-16 (2.15E-10)	5.17E-16 (2.11E-10)	1.30E-13 (1.16E-11)	9.20E-13 (1.32E-11)	1.66E-10 (3.30E-13)	4.98E-12 (1.75E-11)
Patella	2.08E-16 (1.09E-10)	4.47E-16 (1.98E-10)	1.47E-15 (1.66E-10)	9.80E-16 (1.69E-10)	1.10E-13 (1.05E-11)	2.88E-14 (4.55E-11)	4.99E-12 (1.93E-12)	1.20E-08 (3.18E-13)
Remaining bones	2.56E-15 (3.05E-11)	3.02E-13 (1.26E-11)	1.27E-13 (2.25E-11)	6.66E-14 (3.00E-11)	1.52E-13 (1.08E-11)	3.82E-15 (1.25E-10)	1.82E-14 (2.93E-11)	3.46E-15 (4.30E-10)
BM	1.84E-12 (2.04E-12)	2.21E-12 (5.60E-12)	1.86E-12 (6.51E-12)	2.10E-12 (6.35E-12)	1.84E-12 (3.52E-12)	1.67E-12 (1.09E-11)	1.62E-12 (3.61E-12)	1.20E-12 (3.85E-11)

	S [Gy/Bq⋅s] (Relative uncertainty [%])		
	Target organ		
	Remaining bones	BM	Skeleton
	*Source organ*		
Heart	9.75E-14 (1.08E-11)	4.38E-14 (5.73E-12)	5.44E-14 (3.57E-12)
Liver	9.83E-15 (2.46E-11)	2.25E-14 (7.56E-12)	3.37E-14 (4.11E-12)
Lungs	2.03E-14 (2.25E-11)	3.85E-13 (2.18E-12)	3.64E-13 (1.54E-12)
Stomach wall	2.95E-15 (6.77E-11)	8.32E-14 (4.25E-12)	8.76E-14 (2.88E-12)
Pancreas	3.31E-15 (5.88E-11)	3.60E-15 (1.33E-11)	3.54E-15 (9.38E-12)
Kidneys	3.83E-15 (4.45E-11)	5.06E-15 (1.06E-11)	4.61E-15 (7.77E-12)
Spleen	2.72E-15 (5.97E-11)	1.94E-14 (8.43E-12)	1.48E-14 (6.59E-12)
Small intestine	4.19E-15 (4.42E-11)	3.11E-15 (1.38E-11)	3.19E-15 (9.61E-12)
Large intestine	1.37E-13 (8.97E-12)	4.03E-14 (5.98E-12)	3.01E-14 (4.73E-12)
Bladder	6.37E-15 (2.89E-11)	3.19E-15 (1.32E-11)	3.52E-15 (8.74E-12)
Testes	8.14E-15 (5.68E-11)	3.33E-15 (1.35E-11)	4.74E-15 (1.02E-11)
Brain	2.72E-15 (5.06E-11)	4.92E-13 (1.76E-12)	4.09E-13 (1.35E-12)
Thyroid	1.12E-14 (2.62E-11)	1.74E-13 (3.68E-12)	5.01E-13 (1.48E-12)
ROB	1.91E-13 (6.95E-12)	1.15E-13 (3.60E-12)	1.48E-13 (2.11E-12)
Ribs	1.58E-13 (8.59E-12)	1.07E-12 (1.21E-12)	6.79E-12 (3.26E-13)
Spine	1.05E-13 (1.01E-11)	1.40E-12 (1.14E-12)	8.14E-12 (3.06E-13)
Skull	2.14E-15 (5.54E-11)	1.84E-12 (9.20E-13)	7.40E-12 (3.10E-13)
Humerus	6.33E-13 (4.65E-12)	2.21E-12 (8.85E-13)	7.93E-12 (3.17E-13)
Radius	1.78E-13 (1.05E-11)	1.87E-12 (9.05E-13)	7.12E-12 (3.12E-13)
Ulna	6.61E-14 (1.15E-11)	1.77E-12 (9.31E-13)	6.12E-12 (3.16E-13)
Femur	2.05E-13 (7.37E-12)	1.84E-12 (9.21E-13)	7.58E-12 (3.07E-13)
Fibula	3.86E-15 (2.95E-11)	1.54E-12 (9.99E-13)	5.75E-12 (3.23E-13)
Tibia	1.82E-14 (2.09E-11)	1.56E-12 (9.95E-13)	6.22E-12 (3.13E-13)
Patella	3.39E-15 (3.49E-11)	1.10E-12 (1.18E-12)	5.57E-12 (3.05E-13)
Remaining bones	7.93E-11 (3.40E-13)	1.96E-12 (8.85E-13)	6.10E-12 (3.18E-13)
BM	3.80E-12 (4.20E-12)	1.57E-11 (4.26E-13)	8.49E-12 (4.07E-13)

**Table 4:** *S*-values, high resolution (HR) phantom

	S [Gy/Bq⋅s] (Relative uncertainty [%])							
	Target organ							
	Heart	Liver	Lungs	Stomach wall	Pancreas	Kidneys	Spleen	Small intestine
	*Source organ*							
Heart	6.64E-11 (2.49E-13)	2.33E-13 (1.74E-12)	9.64E-13 (1.80E-12)	1.95E-14 (2.75E-11)	8.59E-15 (1.75E-11)	8.45E-14 (6.74E-12)	5.24E-15 (3.42E-11)	8.47E-15 (1.18E-11)
Liver	2.37E-13 (4.13E-12)	1.20E-11 (2.42E-13)	3.93E-13 (3.39E-12)	5.99E-13 (5.62E-12)	1.71E-13 (4.59E-12)	1.23E-13 (5.59E-12)	6.93E-15 (2.81E-11)	6.93E-14 (4.22E-12)
Lungs	9.64E-13 (2.13E-12)	1.58E-13 (2.10E-12)	4.76E-11 (2.54E-13)	1.36E-13 (1.17E-11)	5.08E-15 (2.32E-11)	4.11E-15 (2.17E-11)	4.31E-15 (3.75E-11)	2.89E-15 (1.87E-11)
Stomach wall	1.95E-14 (1.28E-11)	7.14E-13 (1.05E-12)	1.36E-13 (4.65E-12)	2.01E-10 (3.12E-13)	4.58E-12 (1.02E-12)	2.90E-14 (1.03E-11)	2.25E-12 (2.34E-12)	2.16E-13 (2.87E-12)
Pancreas	8.52E-15 (1.50E-11)	5.78E-13 (1.30E-12)	5.02E-15 (1.68E-11)	4.58E-12 (2.29E-12)	9.44E-11 (2.27E-13)	8.66E-13 (2.22E-12)	4.14E-12 (2.01E-12)	9.38E-13 (1.37E-12)
Kidneys	8.44E-14 (6.97E-12)	1.90E-13 (2.12E-12)	4.07E-15 (1.86E-11)	2.89E-14 (2.27E-11)	8.66E-13 (2.17E-12)	6.98E-11 (2.42E-13)	1.67E-13 (8.34E-12)	2.75E-13 (2.59E-12)
Spleen	5.19E-15 (1.92E-11)	7.07E-15 (7.42E-12)	4.30E-15 (1.82E-11)	2.25E-12 (2.90E-12)	4.14E-12 (1.11E-12)	1.67E-13 (4.73E-12)	2.14E-10 (2.45E-13)	5.81E-15 (1.31E-11)
Small intestine	3.90E-15 (1.94E-11)	4.26E-15 (2.19E-12)	1.71E-15 (2.23E-11)	1.24E-13 (1.07E-11)	3.23E-13 (2.30E-12)	8.79E-15 (4.44E-12)	4.00E-15 (3.34E-11)	6.63E-12 (2.48E-13)
Large intestine	3.64E-15 (2.28E-11)	3.98E-14 (4.54E-12)	2.15E-15 (2.57E-11)	1.17E-14 (3.68E-11)	1.77E-12 (1.71E-12)	4.12E-13 (3.31E-12)	6.28E-14 (1.62E-11)	1.03E-12 (1.29E-12)
Bladder	1.31E-15 (3.81E-11)	1.83E-15 (1.51E-11)	9.67E-16 (3.83E-11)	2.11E-15 (6.88E-11)	3.37E-15 (2.87E-11)	3.65E-15 (2.32E-11)	2.54E-15 (5.07E-11)	8.96E-15 (1.01E-11)
Testes	7.48E-16 (5.06E-11)	1.01E-15 (2.03E-11)	5.92E-16 (4.91E-11)	1.14E-15 (9.28E-11)	1.66E-15 (4.08E-11)	1.85E-15 (3.27E-11)	1.37E-15 (6.85E-11)	3.37E-15 (1.66E-11)
Brain	2.84E-15 (2.59E-11)	1.57E-15 (1.57E-11)	3.52E-15 (2.07E-11)	1.33E-15 (8.43E-11)	9.45E-16 (5.39E-11)	8.51E-16 (4.78E-11)	9.60E-16 (8.01E-11)	6.59E-16 (3.93E-11)
Thyroid	7.96E-15 (1.56E-11)	3.05E-15 (1.12E-11)	9.32E-15 (1.29E-11)	2.24E-15 (6.49E-11)	1.59E-15 (4.15E-11)	1.31E-15 (3.85E-11)	1.49E-15 (6.44E-11)	1.07E-15 (3.08E-11)
ROB	7.52E-14 (7.35E-12)	3.71E-14 (4.26E-12)	1.51E-13 (5.47E-12)	1.72E-13 (1.05E-11)	4.98E-14 (8.49E-12)	6.04E-14 (7.94E-12)	6.99E-14 (1.29E-11)	1.15E-13 (3.34E-12)
Ribs	1.51E-13 (5.09E-12)	9.97E-14 (2.75E-12)	1.72E-12 (1.40E-12)	3.63E-13 (7.19E-12)	6.44E-15 (2.06E-11)	5.05E-15 (1.96E-11)	9.56E-14 (1.12E-11)	3.91E-15 (1.62E-11)
Spine	3.02E-14 (1.13E-11)	4.56E-15 (9.18E-12)	3.80E-13 (2.97E-12)	5.54E-15 (4.12E-11)	4.39E-15 (2.44E-11)	7.24E-15 (1.64E-11)	4.81E-15 (3.58E-11)	4.05E-15 (1.54E-11)
Skull	2.39E-15 (2.83E-11)	1.34E-15 (1.70E-11)	2.92E-15 (2.27E-11)	1.12E-15 (9.22E-11)	8.09E-16 (5.82E-11)	7.27E-16 (5.19E-11)	8.16E-16 (8.71E-11)	5.76E-16 (4.20E-11)
Humerus	1.03E-14 (1.36E-11)	4.77E-15 (8.96E-12)	1.54E-14 (1.02E-11)	3.44E-15 (5.26E-11)	2.32E-15 (3.44E-11)	1.93E-15 (3.17E-11)	2.16E-15 (5.33E-11)	1.50E-15 (2.61E-11)
Radius	1.17E-14 (1.28E-11)	9.74E-15 (6.32E-12)	1.53E-14 (9.65E-12)	6.07E-15 (3.98E-11)	4.34E-15 (2.53E-11)	3.24E-15 (2.46E-11)	3.51E-15 (4.21E-11)	2.76E-15 (1.93E-11)
Ulna	1.09E-14 (1.33E-11)	8.89E-15 (6.10E-12)	1.23E-14 (1.77E-11)	5.44E-15 (4.06E-11)	4.09E-15 (2.07E-11)	2.96E-15 (2.51E-11)	3.13E-15 (4.20E-11)	2.66E-15 (1.52E-11)
Femur	8.99E-16 (4.62E-11)	1.21E-15 (1.85E-11)	7.14E-16 (4.50E-11)	1.38E-15 (8.43E-11)	2.06E-15 (3.67E-11)	2.45E-15 (2.85E-11)	1.76E-15 (6.05E-11)	4.09E-15 (1.50E-11)
Fibula	5.16E-16 (6.19E-11)	6.87E-16 (2.22E-11)	4.37E-16 (9.56E-11)	7.64E-16 (1.09E-10)	1.09E-15 (4.06E-11)	1.19E-15 (4.01E-11)	9.10E-16 (7.90E-11)	1.90E-15 (1.81E-11)
Tibia	4.72E-16 (6.41E-11)	6.34E-16 (2.29E-11)	3.99E-16 (9.89E-11)	7.11E-16 (1.13E-10)	9.78E-16 (4.25E-11)	1.05E-15 (4.22E-11)	8.07E-16 (8.29E-11)	1.71E-15 (1.90E-11)
Patella	6.05E-16 (5.66E-11)	8.20E-16 (2.01E-11)	5.08E-16 (8.75E-11)	8.89E-16 (1.01E-10)	1.28E-15 (3.72E-11)	1.38E-15 (3.69E-11)	1.03E-15 (7.35E-11)	2.39E-15 (1.61E-11)
Remaining bones	4.46E-14 (9.42E-12)	4.23E-15 (8.98E-12)	1.08E-14 (1.97E-11)	2.80E-15 (5.65E-11)	3.18E-15 (2.35E-11)	3.65E-15 (2.26E-11)	2.52E-15 (4.67E-11)	4.00E-15 (1.24E-11)
BM	2.11E-14 (1.31E-11)	6.91E-15 (8.76E-12)	3.09E-13 (3.33E-12)	2.50E-14 (2.66E-11)	2.92E-15 (3.04E-11)	3.94E-15 (2.23E-11)	1.30E-14 (2.92E-11)	2.49E-15 (1.98E-11)

	S [Gy/Bq⋅s] (Relative uncertainty [%])							
	Target organ							
	Large intestine	Bladder	Testes	Brain	Thyroid	ROB	Ribs	Spine
	*Source organ*							
Heart	3.69E-15 (2.74E-11)	1.33E-15 (8.36E-11)	7.52E-16 (5.79E-11)	2.94E-15 (2.04E-11)	8.47E-15 (8.64E-11)	7.22E-14 (9.78E-13)	1.52E-13 (6.76E-12)	3.06E-14 (1.09E-11)
Liver	2.46E-14 (1.23E-11)	1.81E-15 (7.17E-11)	1.00E-15 (4.33E-11)	1.52E-15 (2.86E-11)	3.05E-15 (1.25E-10)	3.75E-14 (1.34E-12)	7.93E-14 (8.25E-12)	5.61E-15 (1.73E-11)
Lungs	2.18E-15 (3.57E-11)	9.86E-16 (9.72E-11)	5.95E-16 (6.28E-11)	3.65E-15 (1.83E-11)	9.78E-15 (7.96E-11)	7.81E-14 (1.00E-12)	1.72E-12 (2.23E-12)	3.81E-13 (3.43E-12)
Stomach wall	1.17E-14 (1.86E-11)	2.10E-15 (6.64E-11)	1.13E-15 (4.59E-11)	1.37E-15 (2.99E-11)	2.35E-15 (1.64E-10)	1.77E-13 (6.42E-13)	3.63E-13 (4.50E-12)	5.96E-15 (1.79E-11)
Pancreas	1.77E-12 (1.82E-12)	3.38E-15 (5.25E-11)	1.66E-15 (3.78E-11)	9.72E-16 (3.55E-11)	1.68E-15 (1.94E-10)	1.33E-13 (8.40E-13)	6.37E-15 (2.33E-11)	4.74E-15 (1.99E-11)
Kidneys	4.11E-13 (3.52E-12)	3.66E-15 (5.04E-11)	1.84E-15 (3.74E-11)	8.71E-16 (3.75E-11)	1.35E-15 (2.13E-10)	6.80E-14 (1.04E-12)	5.01E-15 (2.64E-11)	7.89E-15 (1.52E-11)
Spleen	6.29E-14 (9.58E-12)	2.52E-15 (6.07E-11)	1.37E-15 (4.21E-11)	9.86E-16 (3.53E-11)	1.57E-15 (2.00E-10)	7.84E-14 (9.61E-13)	9.53E-14 (8.71E-12)	5.20E-15 (1.89E-11)
Small intestine	3.30E-12 (2.27E-12)	1.31E-14 (3.22E-11)	4.26E-15 (2.69E-11)	4.23E-16 (4.27E-11)	6.66E-16 (2.37E-10)	3.24E-13 (5.99E-13)	2.96E-15 (2.99E-11)	4.46E-15 (2.06E-11)
Large intestine	7.08E-11 (2.70E-13)	1.49E-14 (2.50E-11)	8.53E-15 (3.47E-11)	5.69E-16 (4.64E-11)	9.20E-16 (2.58E-10)	2.74E-13 (5.85E-13)	2.72E-15 (3.57E-11)	6.51E-15 (1.67E-11)
Bladder	1.50E-14 (1.29E-11)	3.27E-10 (2.46E-13)	3.07E-14 (8.99E-12)	3.43E-16 (6.00E-11)	5.04E-16 (3.53E-10)	1.18E-13 (7.72E-13)	1.20E-15 (5.37E-11)	4.43E-15 (2.02E-11)
Testes	8.55E-15 (3.38E-11)	3.07E-14 (1.92E-11)	8.29E-11 (4.81E-13)	2.33E-16 (7.30E-11)	3.44E-16 (4.32E-10)	3.66E-13 (1.19E-12)	7.14E-16 (7.01E-11)	5.44E-15 (2.21E-11)
Brain	5.57E-16 (7.09E-11)	3.30E-16 (1.68E-10)	2.26E-16 (1.04E-10)	4.47E-11 (2.41E-13)	1.74E-14 (5.93E-11)	7.03E-15 (2.68E-12)	2.47E-15 (3.96E-11)	1.47E-14 (1.53E-11)
Thyroid	8.72E-16 (5.65E-11)	4.91E-16 (1.37E-10)	3.22E-16 (8.78E-11)	1.70E-14 (8.54E-12)	1.84E-09 (2.53E-13)	3.30E-13 (5.24E-13)	6.62E-15 (2.44E-11)	9.96E-13 (2.29E-12)
ROB	1.67E-13 (4.97E-12)	1.18E-13 (1.29E-11)	9.30E-14 (6.48E-12)	6.08E-15 (1.68E-11)	1.68E-13 (2.45E-11)	1.22E-12 (2.40E-13)	3.15E-13 (4.52E-12)	2.12E-13 (4.12E-12)
Ribs	2.75E-15 (3.18E-11)	1.22E-15 (8.72E-11)	7.19E-16 (5.93E-11)	2.55E-15 (2.19E-11)	6.95E-15 (9.45E-11)	4.22E-13 (4.26E-13)	7.91E-11 (3.06E-13)	2.59E-13 (4.16E-12)
Spine	6.07E-15 (2.19E-11)	4.11E-15 (4.76E-11)	4.89E-15 (3.91E-11)	1.45E-14 (1.26E-11)	9.98E-13 (1.17E-11)	2.75E-13 (5.46E-13)	2.58E-13 (5.69E-12)	5.22E-11 (2.79E-13)
Skull	4.85E-16 (7.53E-11)	2.98E-16 (1.78E-10)	2.03E-16 (1.13E-10)	1.79E-12 (1.22E-12)	1.41E-14 (6.58E-11)	2.28E-13 (5.60E-13)	2.08E-15 (4.32E-11)	1.89E-13 (4.52E-12)
Humerus	1.19E-15 (4.82E-11)	6.31E-16 (1.21E-10)	4.04E-16 (8.12E-11)	6.52E-15 (1.37E-11)	2.04E-14 (5.49E-11)	3.88E-13 (4.62E-13)	1.25E-14 (2.08E-11)	8.76E-15 (1.89E-11)
Radius	2.04E-15 (3.70E-11)	9.91E-16 (9.72E-11)	6.08E-16 (6.76E-11)	2.48E-15 (2.22E-11)	5.18E-15 (1.09E-10)	2.60E-13 (5.31E-13)	1.61E-14 (1.47E-11)	3.59E-15 (2.50E-11)
Ulna	1.92E-15 (3.19E-11)	9.57E-16 (9.92E-11)	5.84E-16 (5.69E-11)	2.03E-15 (2.49E-11)	4.26E-15 (1.06E-10)	2.25E-13 (5.67E-13)	1.84E-14 (1.25E-11)	3.46E-15 (2.21E-11)
Femur	6.04E-15 (2.12E-11)	1.09E-14 (2.93E-11)	1.17E-14 (1.44E-11)	2.66E-16 (6.84E-11)	3.77E-16 (4.01E-10)	2.26E-13 (5.61E-13)	8.49E-16 (6.44E-11)	5.07E-15 (1.88E-11)
Fibula	2.82E-15 (2.65E-11)	5.93E-15 (3.98E-11)	1.43E-14 (1.15E-11)	1.71E-16 (8.76E-11)	2.36E-16 (4.54E-10)	3.57E-13 (4.50E-13)	6.53E-16 (6.82E-11)	2.86E-15 (2.44E-11)
Tibia	2.34E-15 (2.90E-11)	4.96E-15 (4.37E-11)	1.09E-14 (1.32E-11)	1.58E-16 (9.01E-11)	2.27E-16 (4.65E-10)	1.93E-13 (6.12E-13)	6.02E-16 (7.02E-11)	2.39E-15 (2.67E-11)
Patella	2.80E-15 (2.66E-11)	6.05E-15 (3.95E-11)	8.70E-15 (1.48E-11)	1.92E-16 (8.17E-11)	2.89E-16 (4.12E-10)	2.81E-13 (5.05E-13)	7.77E-16 (6.18E-11)	2.07E-15 (2.87E-11)
Remaining bones	1.18E-13 (5.92E-12)	6.03E-15 (3.95E-11)	5.34E-15 (1.88E-11)	2.40E-15 (2.28E-11)	7.49E-15 (7.98E-11)	2.65E-13 (5.21E-13)	2.55E-13 (5.03E-12)	1.69E-13 (4.59E-12)
BM	2.74E-14 (1.20E-11)	2.65E-15 (5.95E-11)	2.79E-15 (3.66E-11)	9.14E-13 (1.70E-12)	1.77E-13 (2.75E-11)	1.29E-13 (1.14E-12)	2.48E-12 (1.76E-12)	3.06E-12 (1.19E-12)

	S [Gy/Bq⋅s] (Relative uncertainty [%])							
	Target organ							
	Skull	Humerus	Radius	Ulna	Femur	Fibula	Tibia	Patella
	*Source organ*							
Heart	2.47E-15 (3.27E-11)	1.08E-14 (4.81E-11)	1.25E-14 (4.97E-11)	1.16E-14 (5.33E-11)	9.26E-16 (9.41E-11)	6.08E-16 (3.78E-10)	5.79E-16 (1.23E-10)	7.52E-16 (1.04E-09)
Liver	1.59E-15 (3.90E-11)	6.38E-15 (5.55E-11)	1.30E-14 (4.53E-11)	1.21E-14 (4.69E-11)	1.58E-15 (6.63E-11)	9.38E-16 (2.86E-10)	8.38E-16 (9.77E-11)	1.06E-15 (8.53E-10)
Lungs	3.02E-15 (2.96E-11)	1.64E-14 (3.97E-11)	1.64E-14 (4.34E-11)	1.27E-14 (5.15E-11)	7.19E-16 (1.06E-10)	5.03E-16 (4.20E-10)	4.70E-16 (1.37E-10)	6.35E-16 (1.13E-09)
Stomach wall	1.15E-15 (4.81E-11)	3.57E-15 (8.35E-11)	6.46E-15 (6.93E-11)	5.82E-15 (7.61E-11)	1.41E-15 (7.61E-11)	8.78E-16 (3.16E-10)	8.31E-16 (1.03E-10)	1.03E-15 (8.71E-10)
Pancreas	8.35E-16 (5.64E-11)	2.43E-15 (1.02E-10)	4.58E-15 (8.22E-11)	4.39E-15 (8.64E-11)	2.10E-15 (6.25E-11)	1.20E-15 (2.64E-10)	1.15E-15 (8.75E-11)	1.54E-15 (7.44E-10)
Kidneys	7.49E-16 (5.97E-11)	2.02E-15 (1.12E-10)	3.40E-15 (9.53E-11)	3.15E-15 (1.00E-10)	2.49E-15 (5.73E-11)	1.35E-15 (2.53E-10)	1.24E-15 (8.40E-11)	1.71E-15 (6.94E-10)
Spleen	8.34E-16 (5.66E-11)	2.22E-15 (1.06E-10)	3.73E-15 (9.13E-11)	3.40E-15 (9.79E-11)	1.78E-15 (6.78E-11)	1.02E-15 (2.90E-10)	9.68E-16 (9.54E-11)	1.26E-15 (7.98E-10)
Small intestine	4.51E-16 (6.73E-11)	1.24E-15 (1.27E-10)	2.17E-15 (1.03E-10)	2.17E-15 (1.06E-10)	6.82E-15 (4.44E-11)	2.90E-15 (2.01E-10)	2.50E-15 (6.63E-11)	3.51E-15 (5.43E-10)
Large intestine	4.97E-16 (7.32E-11)	1.25E-15 (1.42E-10)	2.15E-15 (1.20E-10)	2.09E-15 (1.30E-10)	6.05E-15 (3.68E-11)	3.16E-15 (1.65E-10)	2.76E-15 (5.64E-11)	3.28E-15 (5.03E-10)
Bladder	3.04E-16 (9.38E-11)	6.51E-16 (1.98E-10)	1.04E-15 (1.73E-10)	1.04E-15 (1.84E-10)	1.11E-14 (2.72E-11)	6.58E-15 (1.14E-10)	5.82E-15 (3.88E-11)	7.38E-15 (3.36E-10)
Testes	2.07E-16 (1.14E-10)	4.22E-16 (2.46E-10)	6.38E-16 (2.21E-10)	6.28E-16 (2.26E-10)	1.20E-14 (2.62E-11)	1.59E-14 (7.33E-11)	1.49E-14 (2.54E-11)	1.05E-14 (2.81E-10)
Brain	1.79E-12 (1.80E-12)	6.57E-15 (6.35E-11)	2.54E-15 (1.11E-10)	2.26E-15 (1.26E-10)	2.66E-16 (1.77E-10)	1.98E-16 (6.56E-10)	1.89E-16 (2.17E-10)	2.53E-16 (1.79E-09)
Thyroid	1.39E-14 (1.39E-11)	2.02E-14 (3.61E-11)	5.26E-15 (7.69E-11)	4.69E-15 (8.20E-11)	3.74E-16 (1.48E-10)	2.86E-16 (5.48E-10)	2.70E-16 (1.82E-10)	3.43E-16 (1.63E-09)
ROB	2.02E-13 (5.08E-12)	2.36E-13 (1.35E-11)	2.24E-13 (1.62E-11)	2.17E-13 (1.64E-11)	1.85E-13 (9.04E-12)	3.43E-13 (2.20E-11)	1.86E-13 (9.69E-12)	2.70E-13 (7.79E-11)
Ribs	2.15E-15 (3.52E-11)	1.31E-14 (4.91E-11)	1.73E-14 (4.17E-11)	1.46E-14 (4.76E-11)	8.70E-16 (9.72E-11)	5.76E-16 (3.85E-10)	5.66E-16 (1.26E-10)	7.39E-16 (1.06E-09)
Spine	1.89E-13 (5.50E-12)	8.83E-15 (6.48E-11)	3.66E-15 (9.23E-11)	3.04E-15 (1.12E-10)	4.74E-15 (4.18E-11)	2.63E-15 (1.82E-10)	2.38E-15 (6.05E-11)	2.11E-15 (6.32E-10)
Skull	6.65E-11 (2.89E-13)	5.41E-15 (7.01E-11)	2.15E-15 (1.20E-10)	1.91E-15 (1.37E-10)	2.33E-16 (1.88E-10)	1.70E-16 (7.12E-10)	1.69E-16 (2.30E-10)	2.40E-16 (1.92E-09)
Humerus	5.39E-15 (2.23E-11)	6.15E-10 (2.90E-13)	3.98E-12 (4.13E-12)	4.29E-12 (3.96E-12)	4.82E-16 (1.30E-10)	3.38E-16 (5.09E-10)	3.31E-16 (1.64E-10)	4.09E-16 (1.44E-09)
Radius	2.09E-15 (3.58E-11)	3.98E-12 (3.53E-12)	7.58E-10 (2.94E-13)	4.16E-11 (1.29E-12)	7.24E-16 (1.07E-10)	5.05E-16 (4.15E-10)	4.70E-16 (1.37E-10)	5.88E-16 (1.19E-09)
Ulna	2.14E-15 (3.38E-11)	3.15E-12 (3.73E-12)	3.07E-11 (1.40E-12)	6.21E-10 (3.03E-13)	9.00E-16 (8.85E-11)	5.47E-16 (3.73E-10)	5.17E-16 (1.25E-10)	7.73E-16 (9.94E-10)
Femur	2.38E-16 (1.07E-10)	5.03E-16 (2.27E-10)	7.43E-16 (2.06E-10)	7.22E-16 (2.10E-10)	2.25E-10 (2.76E-13)	1.48E-14 (7.56E-11)	3.95E-13 (7.01E-12)	2.76E-12 (2.57E-11)
Fibula	1.79E-16 (1.19E-10)	3.79E-16 (2.31E-10)	5.42E-16 (2.27E-10)	5.44E-16 (2.26E-10)	1.75E-14 (1.99E-11)	1.57E-09 (3.20E-13)	1.75E-12 (3.15E-12)	3.47E-14 (1.47E-10)
Tibia	1.67E-16 (1.22E-10)	3.49E-16 (2.38E-10)	5.22E-16 (2.29E-10)	5.09E-16 (2.30E-10)	3.09E-13 (7.00E-12)	1.75E-12 (9.71E-12)	2.05E-10 (2.89E-13)	1.67E-11 (9.99E-12)
Patella	2.03E-16 (1.11E-10)	4.31E-16 (2.16E-10)	9.10E-16 (1.92E-10)	7.82E-16 (1.89E-10)	2.28E-12 (2.62E-12)	3.43E-14 (4.69E-11)	1.67E-11 (1.03E-12)	1.43E-08 (3.28E-13)
Remaining bones	2.45E-15 (3.14E-11)	5.59E-13 (8.84E-12)	2.03E-13 (1.72E-11)	8.07E-14 (2.66E-11)	2.77E-13 (7.41E-12)	3.84E-15 (1.40E-10)	3.86E-14 (2.08E-11)	3.09E-15 (5.05E-10)
BM	3.93E-12 (1.21E-12)	3.81E-12 (3.76E-12)	3.16E-12 (4.63E-12)	3.91E-12 (4.20E-12)	3.09E-12 (2.40E-12)	3.82E-12 (6.99E-12)	2.96E-12 (2.56E-12)	3.36E-12 (2.31E-11)

	S [Gy/Bq⋅s] (Relative uncertainty [%])		
	Target organ		
	Remaining bones	BM	Skeleton
	*Source organ*		
Heart	5.05E-14 (1.32E-11)	2.14E-14 (8.49E-12)	3.35E-14 (4.40E-12)
Liver	5.73E-15 (2.50E-11)	7.02E-15 (1.26E-11)	1.26E-14 (6.08E-12)
Lungs	7.92E-15 (3.15E-11)	3.10E-13 (2.57E-12)	3.61E-13 (1.51E-12)
Stomach wall	2.91E-15 (5.96E-11)	2.52E-14 (8.08E-12)	4.66E-14 (3.83E-12)
Pancreas	3.38E-15 (5.34E-11)	3.11E-15 (1.75E-11)	3.36E-15 (1.07E-11)
Kidneys	4.01E-15 (4.13E-11)	4.30E-15 (1.42E-11)	4.31E-15 (9.05E-12)
Spleen	2.76E-15 (5.34E-11)	1.32E-14 (1.09E-11)	1.59E-14 (6.36E-12)
Small intestine	5.45E-15 (4.07E-11)	2.33E-15 (1.86E-11)	2.96E-15 (1.12E-11)
Large intestine	1.27E-13 (8.28E-12)	2.78E-14 (7.69E-12)	2.32E-14 (5.28E-12)
Bladder	6.58E-15 (2.65E-11)	2.89E-15 (1.71E-11)	3.36E-15 (1.00E-11)
Testes	7.60E-15 (4.67E-11)	3.01E-15 (1.82E-11)	4.00E-15 (1.16E-11)
Brain	2.50E-15 (4.97E-11)	9.14E-13 (1.39E-12)	5.94E-13 (1.09E-12)
Thyroid	9.74E-15 (2.70E-11)	1.78E-13 (3.50E-12)	2.58E-13 (1.90E-12)
ROB	2.56E-13 (5.50E-12)	9.05E-14 (4.36E-12)	1.74E-13 (1.92E-12)
Ribs	3.32E-13 (5.19E-12)	2.48E-12 (8.55E-13)	8.65E-12 (2.87E-13)
Spine	2.15E-13 (6.39E-12)	3.06E-12 (7.99E-13)	1.09E-11 (2.63E-13)
Skull	2.05E-15 (5.60E-11)	3.93E-12 (6.71E-13)	9.87E-12 (2.65E-13)
Humerus	1.24E-12 (2.98E-12)	3.81E-12 (7.04E-13)	1.05E-11 (2.66E-13)
Radius	2.96E-13 (6.27E-12)	3.17E-12 (7.45E-13)	9.68E-12 (2.67E-13)
Ulna	8.04E-14 (9.72E-12)	3.40E-12 (7.19E-13)	8.28E-12 (2.74E-13)
Femur	3.91E-13 (4.77E-12)	3.09E-12 (7.59E-13)	1.04E-11 (2.59E-13)
Fibula	3.85E-15 (3.04E-11)	3.57E-12 (6.99E-13)	7.34E-12 (2.90E-13)
Tibia	3.85E-14 (1.39E-11)	2.88E-12 (7.82E-13)	8.43E-12 (2.71E-13)
Patella	3.03E-15 (3.51E-11)	3.27E-12 (7.29E-13)	7.37E-12 (2.87E-13)
Remaining bones	7.91E-11 (3.10E-13)	4.09E-12 (6.55E-13)	7.99E-12 (2.79E-13)
BM	6.48E-12 (2.46E-12)	2.37E-11 (3.57E-13)	1.17E-11 (3.24E-13)

**Fig. 5:**
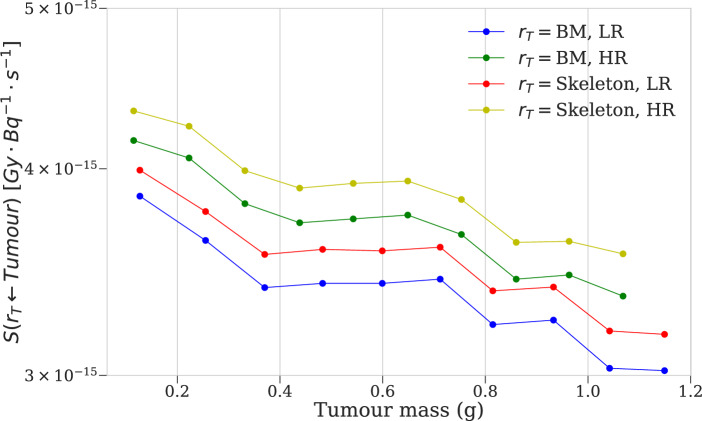
*S*-values targeting mouse bone marrow (BM) and total skeleton from spherical tumours simulated in the left shoulder, as a function of tumour mass. LR phantom: 80 × 80x175 voxels, 0.625 mm cubic voxel size. HR phantom: 140 × 140x355 voxels, 0.29 mm cubic voxel size

**Fig. 6:**
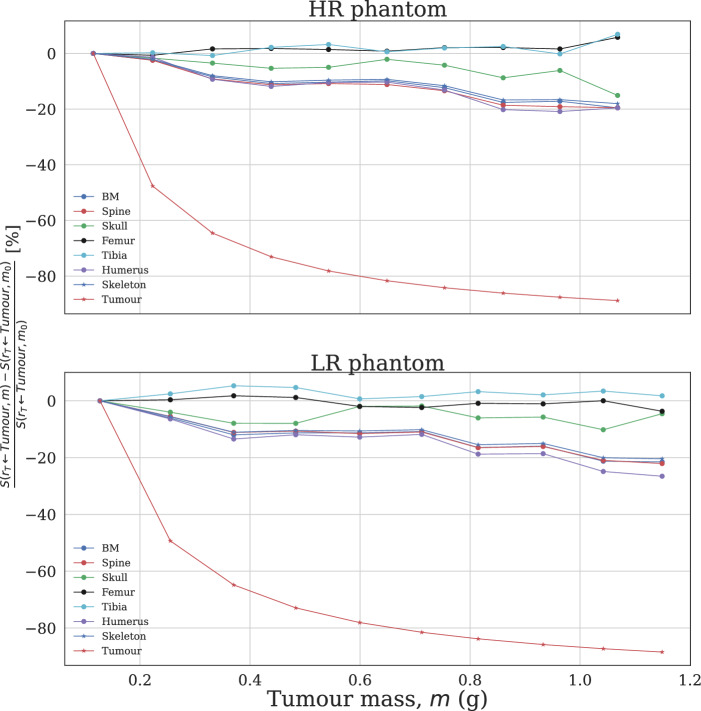
Change in self-dose and cross-dose *S*-values from spherical tumours simulated in the left shoulder, as a function of tumour mass. Smallest tumour mass ${m}_{0}$= 0.12 g at HR, 0.13 g at LR. Top: High-resolution (HR) phantom. Bottom: Low-resolution (LR) phantom

Variation in MOBY phantom resolution did not affect *S*-value magnitude uniformly across all simulations. In tumour simulations, both self- and cross-doses were generally greater at HR, including for the bone marrow (Fig. 5, Table 5). Self-doses were systematically higher at HR, likely attributable to the slightly smaller tumours at this resolution (for instance, the largest tumour weighed 1.07 g at HR, compared to 1.15 g at LR). Recalling the definition of absorbed dose, a similar magnitude of energy deposition divided over a smaller tumour mass is expected to produce a larger self- dose, thereby increasing the corresponding dose factor (Table 5). In the same vein, increasing tumour size for a fixed phantom resolution was observed to decrease the self-dose factor in a fashion similar to exponential decay, in terms of the relative difference from the S-value simulated for the smallest tumour mass (Fig. 6). Cross-doses to the BM and entire skeleton were also systematically greater at HR, likely a product of complex changes in mouse anatomy and beta emission trajectory (as mentioned above and below) and also of smaller tumours increasing the likelihood of beta dose deposition within the mouse body (Fig. 5).

**Table 5:** Tumour self-dose *S*-values for MOBY simulations, with corresponding OLINDA values

Tumour mass (g)	*S*_OLINDA_ (Gy⋅MBq^−1^⋅s^−1^)	*S*_MOBY_ (Gy⋅MBq^−1^⋅s^−1^)	Difference (%)
**0.12**	**1.83E-04**	**1.82E-04**	**−0.69**
*0.13*	*1.66E-04*	*1.64E-04*	**−***1.10*
**0.22**	**9.51E-05**	**9.51E-05**	**−0.01**
*0.26*	*8.33E-05*	*8.30E-05*	**−***0.29*
**0.33**	**6.42E-05**	**6.44E-05**	**0.26**
*0.37*	*5.76E-05*	*5.76E-05*	*0.02*
**0.44**	**4.88E-05**	**4.90E-05**	**0.38**
*0.48*	*4.43E-05*	*4.44E-05*	*0.18*
**0.54**	**3.95E-05**	**3.97E-05**	**0.46**
*0.60*	*3.58E-05*	*3.59E-05*	*0.27*
**0.65**	**3.31E-05**	**3.33E-05**	**0.51**
*0.71*	*3.02E-05*	*3.03E-05*	*0.33*
**0.75**	**2.86E-05**	**2.87E-05**	**0.54**
*0.81*	*2.64E-05*	*2.65E-05*	*0.35*
**0.86**	**2.51E-05**	**2.52E-05**	**0.56**
*0.93*	*2.31E-05*	*2.32E-05*	*0.39*
**0.96**	**2.24E-05**	**2.25E-05**	**0.57**
*1.04*	*2.07E-05*	*2.08E-05*	*0.39*
**1.07**	**2.02E-05**	**2.03E-05**	**0.57**
*1.15*	*1.88E-05*	*1.89E-05*	*0.42*

**HR phantom**: 140 × 140x355 voxels, 0.29 mm voxel size

*LR phantom*: 80 × 80x175 voxels, 0.625 mm voxel size

On the other hand, no general trend in *S*-values was observed for organ-organ simulations; many source-target combinations showed near-parity in values between the two resolutions, while fewer combinations showed greater discrepancies favouring either resolution. Such combinations include all self- and cross-doses to the bone marrow.

Computed *S*-value cross-dose contributions to the BM are relatively low from the pancreas, spleen, bladder, and testes, and higher from the skeleton, brain, lungs, and the rest-of-body (ROB) region. Uniquely, the ROB region, representing ‘background activity’, contains all the remaining mouse tissues after segmentation, and is thus distributed across the body. Cross-dose uncertainties were greatest from the testes, bladder, fibula, tibia, and patella – a mixture of the lowest and greatest contributors of dose, and small and medium-sized tissues (Table 6). Dose uncertainty correlates not only with the dose magnitude, but additionally with the size and geometry of the source tissue, as varying the proximity of the source decay particle varies the number and spatial distribution of particles entering the target organ. The magnitude of cross-irradiation to BM from skeletal structures depends on beta particle energy, penetration range, and material heterogeneity at interfaces like soft tissue, bone, and marrow. For ^177^Lu, the relatively short range of beta particles in tissue confines dose deposition to regions near the emission site. Modeling studies have shown that beta particle transport across these interfaces is influenced by bone density, marrow composition, and the spatial distribution of emitters, which affect energy deposition and dosimetric accuracy. Incorporating these factors into MC simulations, as demonstrated in studies of various alpha and beta emitters including ^177^Lu, is essential to account for dose variations at the bone-marrow interface [66, 68–72]. Comparison of BM *S*-values from MOBY simulations with tumours inoculated in the left shoulder of the mouse provide a degree of insight into BM dose absorption from tumour xenografts in experimental RPT settings. For BM, as for most target tissues, the absorbed dose per unit cumulated activity in the tumours decreases slightly with tumour size. Nonetheless, BM is still one of the components of the skeleton most irradiated by the tumours, along with the spine and skull – owing to the positioning of the tumour in the upper shoulder (Figs. 2 and 5). Although BM is not directly targeted in these simulations, cross-dose contributions from tumours may still be non-negligible, and can vary significantly with tumour burden and location, radionuclide characteristics, and voxel resolution, according to both preclinical and clinical studies [38, 42, 43, 73]. Sandstrom et al. showed that neuroendocrine tumours can contribute up to 30% of BM cross-dose, meanwhile toxicity thresholds of 2 Gy per cycle are typically observed, beyond which patients risk experiencing acute toxicity and suppression [26, 42]. The cross-dose from subcutaneous tumours is generally lower than in models where disease is localized to or infiltrates the bone, as seen in metastatic prostate cancer models [74]. Even in these subcutaneous tumour models, however, BM receives measurable radiation exposure, demonstrating the need for accurate BM dose estimates when interpreting potential hematopoietic toxicity [47, 75].

**Table 6:** S-values targeting BM for various source organs, compared to corresponding values from Tamborino et al. [41]

Source organ	*S*(BM ← r_s_) [Gy⋅Bq^−1^⋅s^−1^]				
	Tamborino et al.	LR phantom	Difference (%)	HR phantom	Difference (%)
Liver	1.11E-14	1.66E-14	*49.7*	3.16E-15	*−71.6*
Kidneys	3.52E-15	3.74E-15	*6.2*	1.93E-15	*−45.1*
Spleen	1.53E-14	1.43E-14	**−***6.6*	5.92E-15	*−61.4*
BM	8.40E-11	1.16E-11	**−***86.2*	1.07E-11	*−87.3*

The use of italics is to distinguish the columns with 'relative difference' data from those with measures of S-values or organ masses

This study is limited in scope with respect to two factors. First, *S*-values were computed for a single ‘standard’ mouse anatomy, including a single distribution of BM taken from the MOBY phantom. In future, variation in marrow distributions between mice could be considered using methods such as ^99m^Tc-labelled sulfur colloid SPECT-CT imaging, and dose rates for BM could be estimated for segmented skeletal regions, as demonstrated by Tamborino et al. and other groups [41, 76]. Additionally, variations in other parameters, such as mouse mass and respiratory or cardiac motion were not explored. Most critically, the MOBY phantom allows for simulation of only male mice, whereas previous work has shown differences in male and female mouse anatomies are significant enough to produce non-negligible differences in *S*-values for select organs, including for ^177^Lu [77]*.* Secondly, our scope is limited in that only ^177^Lu was considered in the computation of *S*-values. This radionuclide currently predominates RPT development in both the preclinical and clinical phases, with recent radiopharmaceuticals FDA-approved using it [2–4, 7, 28]*.* Thus, the herein presented results are intended to support the preclinical development of novel ^177^Lu-labelled radiopharmaceuticals as well as to support the understanding of the currently approved radiopharmaceutical therapies. However, future studies should aim to simulate *S*-values for other radionuclides, which may have different BM toxicity profiles based on their particle ranges and ionizing energies. For instance, the toxicity profile of ^161^ Tb, which has emerged as a promising candidate for clinical translation in RPT, has been shown by Hemmingsson et al. to be more dependent on the radionuclide distribution within the marrow cavity than that of ^177^Lu, owing to its relatively larger beta-particle energy emissions [36]. Elsewhere, radionuclides such as ^225^Ac, ^221^At and ^223^Ra, whose alpha emissions have shorter ranges and higher linear energy transfers, may enable precise, localized dosing with reduced marrow toxicity [71, 74, 78, 79]. These findings warrant increased consideration of alpha-emitting radionuclides in the preclinical phase, including through accurate computation of BM *S*-values.

Standardizing preclinical dosimetry models is essential to ensure consistency in *S*-value calculations, enabling reliable dose–response predictions and facilitating clinical translation of radiopharmaceutical therapies [60]. Differences in organ mass estimation and tissue density can significantly alter absorbed dose calculations, highlighting the need for harmonized methodologies [61]. While anatomically realistic hybrid phantoms improve accuracy, they alone do not ensure universally applicable dosimetric data [24]. Standardized Monte Carlo-based approaches, particularly for bone marrow dosimetry, enable more accurate translation of preclinical findings to human applications [55, 80]. Establishing consensus on dosimetry methods will improve cross-study comparability, support regulatory evaluation, and advance safer, more effective RPTs [12].

The findings of this study aim to support clinical translation by refining BM dosimetry in preclinical models. The observed influence of tumour burden on BM cross-dose suggests that incorporating tumour load into patient-specific dosimetry calculations could improve toxicity predictions, while the influence of voxel resolution demonstrates the need for high-resolution clinical imaging to minimize uncertainty in radionuclide therapy [12, 16, 26, 61, 75]. Standardized preclinical dosimetry methods are essential for bridging preclinical and clinical RPT, particularly as MIRD-based approaches continue evolving toward patient-specific and voxel-level dosimetry [9, 14]. Addressing these factors could improve BM dose estimates in clinical RPT, contributing to better risk assessment and treatment planning [32, 46].

## Conclusion

This work presents BM organ-organ *S*-values for the MOBY digital mouse phantom, including *S*-values for inoculated tumours in the shoulder, for ^177^Lu radiopharmaceuticals. We hope that the publication of these values contributes toward advancing radiopharmaceutical development and improving preclinical studies. By providing more accurate preclinical BM dose estimates, these findings aim to improve dosimetry-based toxicity assessments, aid in optimizing patient-specific treatment planning, and guide the selection of novel radiopharmaceuticals for clinical translation, particularly in therapies where BM toxicity is a dose-limiting factor.

## Supplementary Information

Additional file1 (XLSX 10 KB).

Additional file 2 (DOCX 12 KB).

## Data Availability

Data and code available upon request to the corresponding author, or at Zenodo, 10.5281/zenodo.6812525.

## Abbreviations

BM: Bone marrow
CT: Computed tomography
GATE: Geant4 application for tomographic emission
MC: Monte Carlo
MOBY: 4D mouse whole body
OLINDA: Organ level internal dose assessment
PET: Positron emission tomography
RADAR: Radiation dose assessment resource
RPT: Radiopharmaceutical therapy
SAF: Specific absorbed fraction
SPECT: Single photon computed emission tomography
