# Supplementary material for: S-values for bone marrow dosimetry in preclinical radiopharmaceutical studies with rodents
Source: EJNMMI Phys. 2025 Jul 8;12:67. doi: 10.1186/s40658-025-00752-5 (PMC12238459; doi:10.1186/s40658-025-00752-5)
Supplement: Supplementary file 2 — Additional file 2 (DOCX 12 KB). [file 40658_2025_752_MOESM2_ESM.docx]

**Supplemental Tables**

**Table A1. Full list of segmented tissues and corresponding masses for MOBY mouse phantoms simulated in this study.**

Low-resolution phantom parameters: 74x74x184 voxels, 0.625 mm voxel size.

High-resolution phantom parameters: 128x128x400 voxels, 0.29 mm voxel size.
